# White matter trajectories over the lifespan

**DOI:** 10.1371/journal.pone.0301520

**Published:** 2024-05-17

**Authors:** Stefania Conte, Dabriel Zimmerman, John E. Richards

**Affiliations:** 1 Department of Psychology, State University of New York at Binghamton, Vestal, NY, United States of America; 2 Department of Biomedical Engineering, Boston University, Boston, MA, United States of America; 3 Department of Psychology, University of South Carolina, Columbia, SC, United States of America; University of North Carolina at Chapel Hill, UNITED STATES

## Abstract

White matter (WM) changes occur throughout the lifespan at a different rate for each developmental period. We aggregated 10879 structural MRIs and 6186 diffusion-weighted MRIs from participants between 2 weeks to 100 years of age. Age-related changes in gray matter and WM partial volumes and microstructural WM properties, both brain-wide and on 29 reconstructed tracts, were investigated as a function of biological sex and hemisphere, when appropriate. We investigated the curve fit that would best explain age-related differences by fitting linear, cubic, quadratic, and exponential models to macro and microstructural WM properties. Following the first steep increase in WM volume during infancy and childhood, the rate of development slows down in adulthood and decreases with aging. Similarly, microstructural properties of WM, particularly fractional anisotropy (FA) and mean diffusivity (MD), follow independent rates of change across the lifespan. The overall increase in FA and decrease in MD are modulated by demographic factors, such as the participant’s age, and show different hemispheric asymmetries in some association tracts reconstructed via probabilistic tractography. All changes in WM macro and microstructure seem to follow nonlinear trajectories, which also differ based on the considered metric. Exponential changes occurred for the WM volume and FA and MD values in the first five years of life. Collectively, these results provide novel insight into how changes in different metrics of WM occur when a lifespan approach is considered.

## 1. Introduction

Magnetic resonance imaging (MRI) can be used to track in vivo developmental changes in brain structure across the lifespan. Different head tissues mature following specific timelines and have a different impact on behavioral and cognitive changes. Much research has been conducted to detail the brain changes from childhood to adulthood. Recently, data collection protocols and acquisition sequences have been adapted to young infants and allowed us to obtain important information on structural brain development in younger subjects. However, very few studies have performed analyses considering a lifespan perspective in which structural changes are investigated from early in life [1]. To the best of our knowledge no study has implemented a lifespan approach to investigate macro and micro structural changes in white matter (WM) through probabilistic tractography. The goal of the current study was to investigate the effect of age on structural brain changes with particular reference to the development of WM. We used data from open-source MRI datasets and compared different model fits to identify the development of the whole-brain and tract-based WM volume, fractional anisotropy (FA), and mean diffusivity (MD). We tested whether the developmental changes followed a different path in female and male participants and whether there were hemispherical differences in WM growth.

Much research has been conducted to investigate the development and role of gray matter (GM), overlooking changing aspects of WM that may be critically involved in behavioral and cognitive processes [2]. According to such a localizationist approach, cognitive functions are identified in isolated cortical areas using one-to-one mapping principles and little consideration for the underlying connectivity. A more recent framework identifies anatomo-functional couplings that dynamically interact in the formation of specialized networks as the basis of complex cognitive functions and behaviors [3]. Thus, the cortical organization is integrated with information on the structural connectivity of WM fiber bundles. Diffusion tensor imaging (DTI) provides an in vivo investigation of WM fiber bundles in a noninvasive fashion in humans, allowing us to study the spatial organization of cerebral tissues [4]. The role of WM bundles in sustaining and ensuring rapid and efficient neuronal interactions has been described for perceptual (e.g., vision and sensorimotor processes) and cognitive processes (e.g., memory and language) and with limited extension to social cognition [5]. Even fewer studies investigated the relation between WM and cognition in early development as the majority of evidence comes from studies of aging. Nonetheless, diffused WM injuries seem to be linked to neurodevelopmental and pediatric neurocognitive disorders [6, 7], the incidence of which is growing in recent years [8]. The organization of structural and functional networks is interconnected, with structural networks influencing the dynamic properties of functional networks at a faster pace than the reverse interaction [9]. Given these relationships, it seems critical to detail the lifespan development of anatomical connectivity and changes in WM properties.

Acquisition sequences and protocols have been adapted to participants with different ages and characteristics to provide quantitative parameters of both typical and atypical neurodevelopment. Despite variability in the methods, there are consistent findings about cerebral WM development. Neurodevelopmental changes can be related to macrostructural and microstructural characteristics of WM. Macrostructural characteristics refers to volumetric changes in WM that occur at a different rate and with different regional distributions across the lifespan. A good quantification of WM volume can be obtained through structural MRI segmenting procedures and investigated in relation to the development of cortical areas involved in both sensory and association processes.

Microstructural characteristics of WM quantifies tissue properties and integrity using metrics that measure the apparent diffusion coefficient of water. Diffusion-weighted imaging (DWI) sequences are designed to estimate axonal orientation in vivo by measuring the degree of water diffusion along specific directions (i.e., anisotropy). Diffusion tensor (DT) models are the simplest way to obtain information on the properties of WM, which are expressed with different metrics (e.g., mean diffusivity, MD, fractional anisotropy, FA). WM integrity is often measured through FA, whereas MD, axial and radial diffusivity (AD, RD) are indicators of WM maturation and dysfunction [10, 11]. DTI metrics can also be used to reconstruct specific fiber bundles using tractography methods that allow the identification of the major WM tracts and shorter connections between cortical areas. Microstructural WM properties could also be investigated via multi-compartment models of structural MRI or specific quantitative MRI methods, e.g., magnetization transfer [11, 12].

A rapid increase in volume and WM metrics change occur in the first years of life and continues through the lifespan [13]. Morphometry studies based on structural MRI showed that changes in brain volume during childhood and adolescence are as dramatic as the changes occurring later in life. The increase in both GM (cortical and subcortical) and WM partial volumes during childhood and early adulthood gives way to a decrease later in life. Developmental trajectories of GM and WM follow different timelines, with GM increasing more rapidly, whereas WM changing gradually in the first two years of life [14]. The volume of frontal and temporal lobes shows rapid changes in the first two years of life, possibly due to maturational processes of neuronal pruning [15, 16]. Although most of the studies focused their investigation on developmental changes in the middle portion of the lifespan (i.e., childhood, adolescence, and adulthood), important changes occur at both early and late developmental stages [17–20]. Specifically, the developmental trajectory of brain growth in infancy seems to occur along non-linear patterns that modify the quadratic trend usually used to describe later changes [21, 22]. For this reason, and because the availability of structural MRI and DWI datasets has reached a consistent sample size only in recent years, a comprehensive lifespan investigation remains poorly studied.

Changes in microstructural WM properties seem to dramatically increase in the first year of life, slowly change during the second year of life, and stabilize after 24 months. It has been estimated that FA increases by about 31% between the first and second and third years of life, whereas only a 6% increase occurs later in childhood [23]. Almost all major WM bundles are identifiable at birth and show to increase in size and FA over the ages. Specifically, FA values in peripheral regions of the tracts are reported to be low and similar to GM until 3 months of life, whereas higher FA values are identified in the core areas of fiber bundles early in life [24]. Projection, limbic, and callosal fibers can be identified in newborns. On the other hand, association tracts, such as the superior longitudinal, inferior fronto-occipital, and inferior longitudinal fasciculi are hard to detect in newborns but become visible within the first year of life [24]. The pattern of FA increase reaches its maximum in long projection first, then commissural, and finally association fibers, which show age-related FA changes until adulthood [25, 26].

Several statistical models have been implemented to define the best fit to explain age-related changes in different brain tissues. Such modeling approaches have been applied to variable sample sizes of MRI acquisitions from different age ranges. Although non-linear models have been proven to best explain brain changes, many studies had addressed the issue by utilizing linear approaches (for a review, see [27]). A more recent approach uses generalized additive models to define growth charts for brain changes. These analyses have provided detailed trajectories of typical development and identified brain growth milestones specific for each brain tissue. Total brain volume is reported to peak at 10–12 years of age and show biological sex differences with males having larger age-adjusted total brain volumes than females, regardless of participants’ body size [28]. Negative associations are found between cortical thickness and age, with the highest thickness values identified during childhood for most of the cortical regions, even though frontal and temporal areas showed the highest interindividual variability [29]. Similarly, the volume of most subcortical regions peaks at 2–3 years of age and keeps growing for some regions, such as the hippocampus, amygdala, and putamen [30]. The introduction of standardized charts of brain change provides a useful tool to identify developmental milestones and associate normative neurodevelopmental trajectories with neuropsychiatric disorders [1].

In the current study, we investigated the WM changes in the lifespan by combining structural MRI and DWI acquisitions from participants ranging from 2 weeks to 100 years of age. The goal of the study is to investigate the developmental trajectories of the WM volume and fiber tracts and their microstructural properties, considering the entire lifespan. We applied different model fits (linear, quadratic, cubic, and exponential) to quantify the age-related changes in WM partial volume and its microstructural properties (i.e., FA and MD). We further investigate possible interactions between age and biological sex and hemisphere to the same WM metrics. Lastly, we applied probabilistic tractography algorithms to reconstruct 16 WM bundles and tested the change in each WM metric as a function of age, biological sex, and, when appropriate, hemisphere.

## 2. Materials and methods

### 2.1 Subjects

The MRI volumes for this study came from local scans and open-access sources as available in January 2022. The Institutional Review Board of the University of South Carolina approved all procedures, which were conducted according to the Declaration of Helsinki. Written informed consent form was obtained from all subjects scanned at the University of South Carolina. The open-access sources included deidentified data and volumes from the Human Connectome Project (https://www.humanconnectome.org/): HCP [31], HCP-development [32], HCP-aging [33], and BabyCP [34]. The USC-ABC [35] and other local scans acquired at the McCausland Center for Brain Imaging (MCBI) used the HCP sequence. Other sources include the infants from the IBIS [36, 37] and UNC-EBDS [38–40], children from PING [41], BAMBAM [42], and CMIHBN, and adults from OASIS [43], CMIHBN [44], and CAMCAN [45, 46].

Structural MRI volumes were available for 10879 subjects, from 1 day to 100 years of age. We excluded 366 participants because of missing information about their biological sex. The remaining 10513 participants (*n* = 5569 females, *n* = 4944 males) were considered for further analyses. Diffusion MRI volumes were available for a subset of 6189 participants, 3 of whom were excluded because of missing information about their biological sex. Volumes from the UNC-EBDS and volumes acquired with single-shell sequences were excluded (*n* = 782). A total of 5407 participants (*n* = 2864 females, *n* = 2540 males), ranging from 30 days to 89 years, were considered for further analyses.

**Fig 1:**
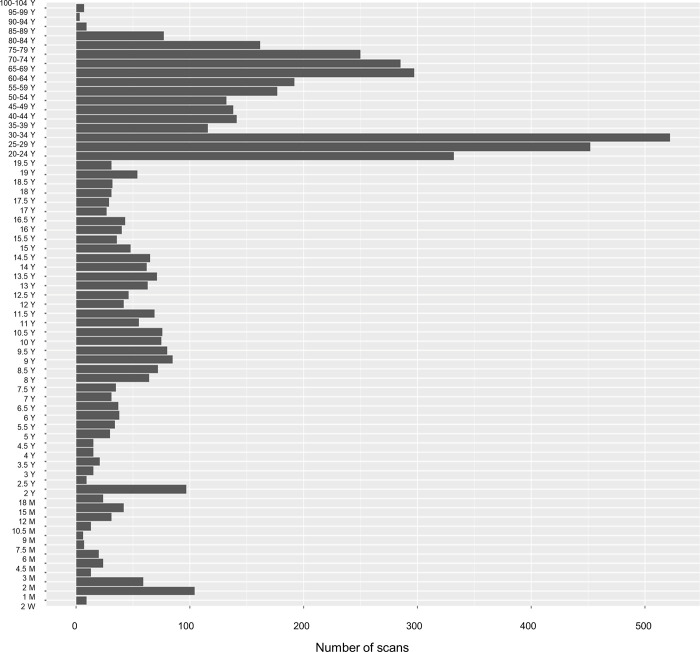
Number of structural scans per age group.

**Fig 2:**
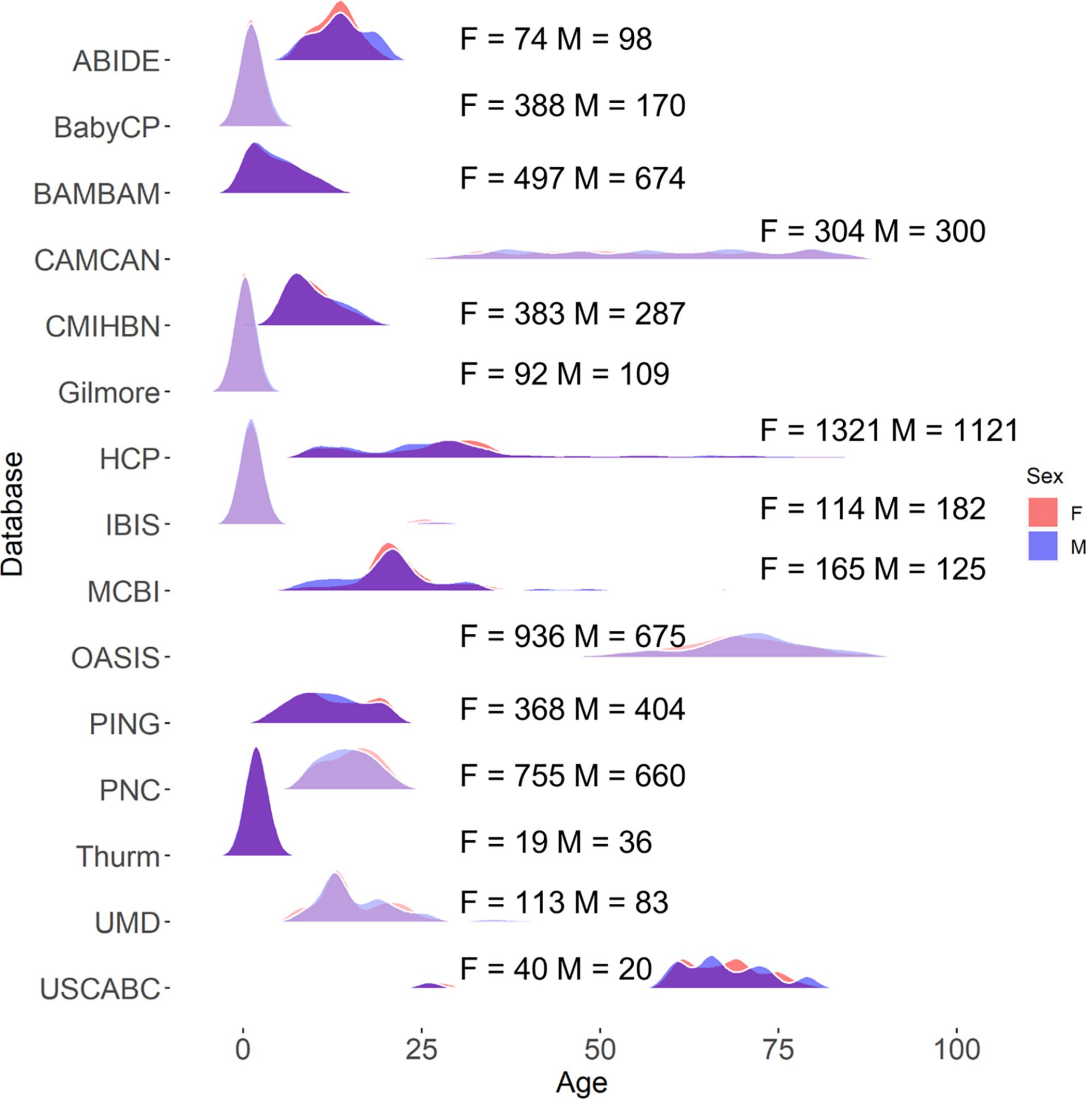
Distribution participant as a function of biological sex for each considered database.

Fig 1 summarizes the number of participants per database and Fig 2 the distribution of female and male subjects in each database. Additional information can be found in S1 Table. Deidentified data is available at https://www.nitrc.org/projects/neurodevdata.

### 2.2 Image acquisition protocols and preprocessing

Details on the acquisition protocols for the different open-source databases included in the current sample can be found in the respective publications and are summarized in Table 1. Image preprocessing was performed using FSL tools using the same procedures across all volumes. All available T2-weighted volumes were registered to the T1-weighted images through affine transformation and used to register DWI volumes to structural volumes for visualization purposes. Transformation matrices of individual structural volumes and the age-appropriate templates were obtained through FLIRT and ANTS. Skull stripping was performed utilizing brain extraction steps included in FSL-VBM [47] and outputs were visually inspected for brain extraction accuracy and manually corrected if needed. Volume segmentation was performed with FSL-FAST [48]using the T1 image to obtain gray and white matter partial volumes. The procedure was guided by the segmented volumes from the age-appropriate average templates for subjects of 2 years and younger. Binary masks of the WM partial volume were also created and used to restrict the analyses on the diffusion data (see *Structural and Diffusion Measurements* for details).

**Table 1:** T1-weighted and T2-weighted spatial resolutions and DWI b-values for each considered database. Further details on the acquisition protocols can be found in the respective publications.

Database	T1-w (spatial resolution in mm)	T2-w (spatial resolution in mm)	DWI non-zero b-values
HCP	1x1x1	1x1x1	500 to 10,000 s/mm^2^
HCP-development	0.8×0.8×0.8	0.8×0.8×0.8	1500 and 3000 s/mm^2^
HCP-aging	0.8×0.8×0.8	0.8×0.8×0.8	1500 and 3000 s/mm^2^
BabyCP	0.8×0.8×0.8	0.8×0.8×0.8	400, 1000, 2600 s/mm^2^
			700, 1500, 3000 s/mm^2^
			500, 1000, 1500, 2000, 2500, 3000 s/mm^2^
USC-ABC	1x1x1	0.9x0.9x0.9	2000 s/mm^2^
IBIS	1x1x1	1x1x1	maximum of 1,000 s/mm^2^
UNC-EBDS	1x1x1	1.25x1.25x1.50	na
PING	1x1x1	1x1x1	1000 s/mm^2^
BAMBAM	1x1x1	1.8×1.8×1.8	na
CMIHBN	1x1x1	0.9×0.9×5.0	1000, and 2000 s/mm^2^
OASIS	1x1x1	1x1x1	1000, and 2000 s/mm^2^
CAMCAN	1x1x1	1x1x1	1000, and 2000 s/mm^2^

Diffusion data was preprocessed using the FMRIB’s Diffusion Toolbox (FDT) in FSL [49]. This procedure estimates and corrects for susceptibility-induced distortions using volumes acquired with reversed-phase encoding directions (*topup*, [49, 50]), Eddy current, and motion artifacts (*eddy_cuda*, [51]). Single-band reference (SBref) volumes are utilized to register EPI images to the T1 volume. Eddy current and motion corrections were performed on uncorrected images when only one phase-encode blip was acquired. Diffusion tensor models were calculated at each voxel to obtain FA and MD measures (*ditfit*).

### 2.3 Tractography

Thirteen lateralized (26 total) and three callosal tracts were reconstructed using probabilistic tractography models. Distributions of diffusion parameters were calculated using *BEDPOSTX* at each voxel [52]. Seed, target, and exclusion masks included in the tractography (XTRACT) toolbox [53, 54] were first registered to the age-appropriate template selected from the Neurodevelopmental MRI Database [55] and then to the individual volume using FLIRT. This set of masks was utilized to obtain connectivity matrices using the *BEDPOSTX* estimations via probabilistic tractography (*PROBTRACKX*, [56, 57]. We set a relative threshold of 5% of the total number of valid streamlines (waytotal) to generate a mask for each reconstructed tract [58], which were then used to restrict the computations of the white matter properties of each tract (see section below). Procedures resulting in zero waytotal values were not considered for further the analyses.

### 2.4 Structural and diffusion measurements

We calculated average GM and WM volumes for the individuals with available structural MRI data. All structural measures were based on T1 volumes. Tools in FSL were utilized to define all segment types. We calculated normalized volumes by dividing the GM and WM partial volumes by the total brain size of each subject. We reported results for the uncorrected and normalized volumes.

Brain-wide FA and MD resulting from the diffusion tensor fitting (*dtifit*) of diffusion MRI were masked with the WM volume before averaging. Similarly, average FA, MD, and density values were obtained for each selected tract.

### 2.5 Analytic strategy

Outliers were defined as the values above the 75^th^ or below the 25^th^ percentile of the distribution of each database and measure (GM: *n* = 120; WM: *n* = 78; FA: *n* = 136; MD: *n* = 266). All analytical strategies were applied to the full sample and a subset of participants ranging from 0 to 5 years. The latter analyses would shed light on the differences across years that are usually overlooked or underrepresented in studies investigating brain development in the lifespan.

Age-related changes of all structural and diffusion MRI measurements were analyzed using mixed-effect models. A hierarchical approach was utilized to test the effect of age, biological sex, and their interaction, considering the database as a random factor for all measures to consider the variability generated by the different sets of acquisition parameters. We did not do specific preprocessing harmonization across the different datasets, but instead used database as a random factor in our analyses to account for inter-database variability (for a similar approach, see [59–62]). Several novel pipelines for data harmonization have been recently developed and successfully normalize data, retaining enough variation at the level of individual scans (e.g., ComBat, [63]; ComBat-GAM, [64]; GAMLSS, [1, 65]). However, these pipelines require volume normalization to an adult template as a preparatory step. This approach would introduce a bias in the registration process of volumes that are volumetrically different than the target template, therefore introducing more noise at the extremes of the lifespan than in volumes that are registered to an age-appropriate template. Harmonization procedure such as ComBat-GAM showed to perform equally well to alternative harmonization methods (i.e., linear-mixed effect modeling, ComBat), after removing data from sites with participants younger than 20 years and older than 70 years [66]. For all analyses we fitted models with and without Database as random factor and found an improvement in the model fit when Database was considered. Further information on this set of analyses can be found in the Supplementary Materials (S2 Table, S1 Fig). All results reported here include Database as random factor to control for unwanted variability introduced by technical differences across scanners. This approach also avoids data normalization to a single, not age-appropriate, template. We introduced the fixed effect of hemisphere in the tract analyses when appropriate (i.e., for all tracts except the three sections of the corpus callosum).

In a separate set of analyses, we compared the null (i.e., with the intercept term only), linear, quadratic, cubic, and exponential fits for all structural and diffusion MRI measures. The following equations were fit for the data: measure ~ 1 (1 | Dataset, participant); measure ~ Age + (1 | Dataset, participant); measure ~ Age + Age^2^ + (1 | Dataset, participant); measure ~ Age + Age^2^ + Age^3^ + (1 | Dataset, participant); measure ~ Age + Age^2^ + Age^3^ + Age^ (1 | Dataset, participant). ANOVAs were performed to compare the different model fits.

## 3. Results

### 3.1 Structural measures

**Fig 3:**
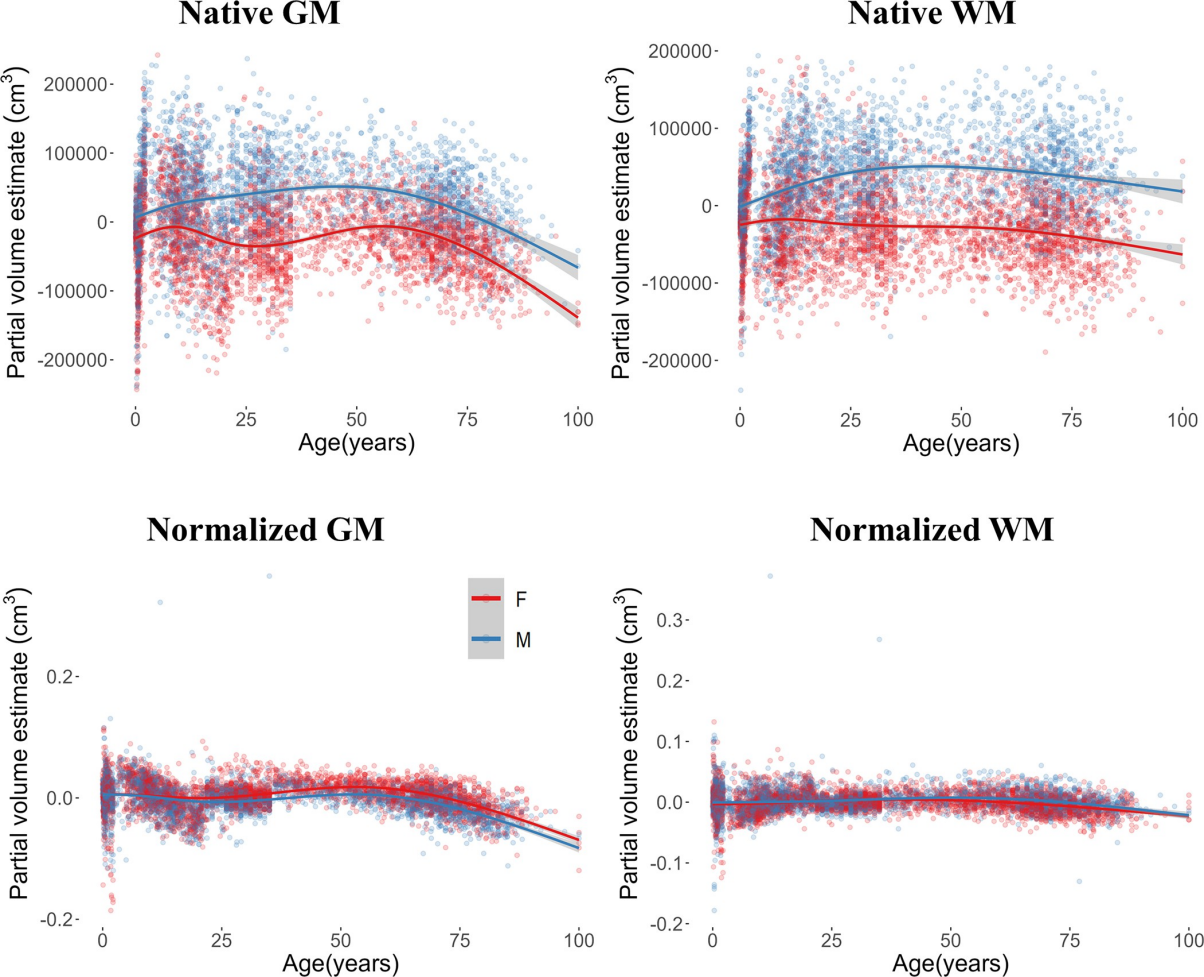
Uncorrected and normalized GM and WM volumes as a function of biological sex across the lifespan. Plotted values represent the residuals of a null model for each measure.

The GM and WM volume changes across the lifespan are reported in Fig 3 as residual values of the null model in which the database factor was accounted for. Both measures showed larger values for males than females in the uncorrected volumes. This difference did not occur when the GM and WM were corrected for the participants’ total brain volumes. Results of the uncorrected GM volume showed a relation to the interaction effect of Age and Sex (beta = .564, *F*(1,10226) = 53.43, *p* < .001), to the main effect of Age (beta = -1.742, *F*(1, 10226) = 398.56, *p* < .001), and Sex (beta = 60.404, *F*(1, 10226) = 436.70, *p* < .001). After controlling for the total brain volume, the corrected GM values were related to the interaction effect (beta = -0.0002, *F*(1,10226) = 50.07, *p* < .001) and to the Age (beta = -0.0011, *F*(1,10226) = 587.23, *p* < .001). The relation between the normalized GM values and Sex was nonsignificant (beta = -0.0004, *F*(1,10226) = .246, *p* = .62). Similarly, the uncorrected WM volume was related to the interaction effect of Age and Sex (beta = 0.737, *F*(1,10268) = 123.40, *p* < .001), to Age (beta = -0.48, *F*(1, 10268) = 10.35, *p* = .001), and to Sex (beta = 49.48, *F*(1, 10268) = 395.50, *p* < .001). Results on the normalized WM volumes showed that, after correcting for total brain volume, there were reduced differences between female and male WM values (Sex: beta = -0.001, *F*(1, 10268) = 4.37, *p* = .037). White matter volume declined over age (beta = -.0005, *F*(1, 10268) = 132.29, *p* < .001) and showed to be related to the interaction effect (beta = .0001, *F*(1, 10268) = 31.65, *p* < .001).

We tested four model fits to describe the age changes in GM and WM across the lifespan. Specifically, adjusted GM and WM values were tested for the linear, quadratic, cubic, and exponential age patterns. The exponential model best described the development of the average GM and WM volumes (GM: AIC = 264285.5; beta = 6.153, *F*(1,10226) = 4810.85, *p* < .0001; WM: AIC = 262525.5; beta = -0.524, *F*(1,10267) = 2924.89, *p* < .0001), with a significant improvement than the linear (GM: AIC = 26887.9, *p* < .0001; WM: AIC = 266261.2, *p* < .0001), quadratic, (GM: AIC = -268651.6, *p* < .0001; WM: AIC = 265800.5, *p* < .0001) and cubic fits (GM: AIC = 268181.9, *p* < .0001; WM: AIC = 265094, *p* < .0001).

**Fig 4:**
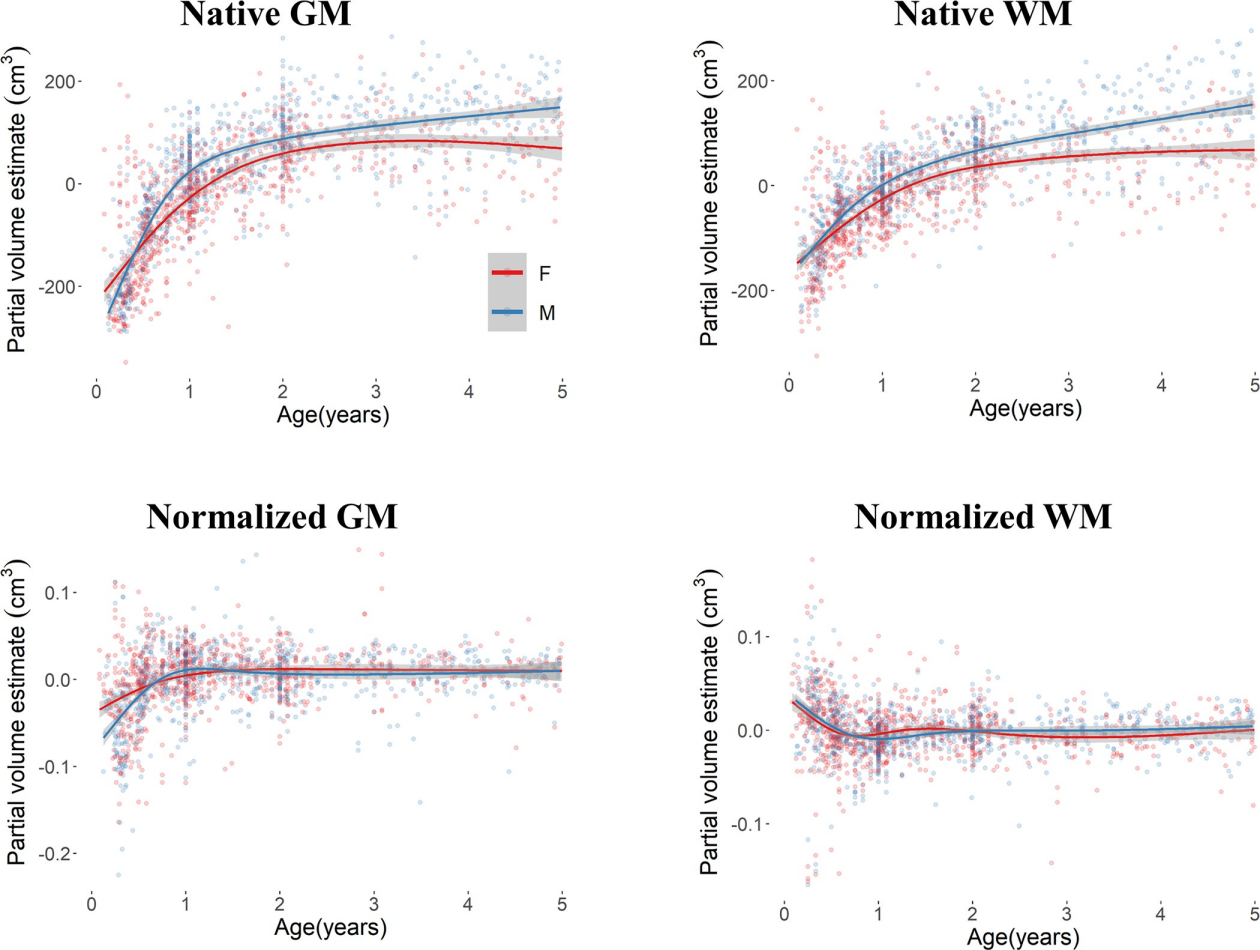
Residual values of the null model for uncorrected and normalized GM and WM volumes as a function of biological sex for the first 5 years of life.

Changes in GM and WM over the first 5 years of life (*n* = 1656; outliers_GM_ = 14; outliers_WM_ = 7) are reported in Fig 4 for uncorrected and corrected volumes as a function of biological sex. Results showed a relation between the GM change and both Age (beta = 95.02, *F*(1,1646) = 228.37, *p* < .001) and Sex (beta = 41.78, *F*(1,1646) = 31.27, *p* < .001) factors but not their interaction (*p* = .082). After correcting for brain volume, the GM change showed to be related to the Age factor only (beta = 0.014, *F*(1,1646) = 19.50, *p* < .0001). In the first five years of life, the GM volume increased for both female and male participants. Results on uncorrected WM volumes showed a relation to the interaction effect of Age and Sex (beta = 12.57, *F*(1, 1652) = 25.82, *p* < .0001), to the main effects of Age (beta = 60.04, *F*(1, 1652) = 215.74, *p* < .001), and Sex (beta = 25.93, *F*(1, 1652) = 28.73, *p* < .001). After adjusting for brain volume the WM change was related to the Age factor only (beta = -0.0115, *F*(1, 1652) = 32.82, *p* < .001) and showed an overall decrease in the first 5 years.

When tested over the four model fits the GM and WM development over the first 5 years of life showed to be best described by the exponential model (GM: AIC = 46132.5; beta = 1.16, *F*(1,1646) = 8.900, *p* = .003; WM: AIC = 45341.67; beta = 5.11, *F*(1,1652) = 15.09, *p* = .0001), with a significant improvement than the linear (GM: AIC = 46690.7, *p <* .0001; WM: AIC = 45635.68, *p* < .0001), quadratic (GM: AIC = 46181.2, *p* < .0001; WM: AIC = 45384.57, *p* < .0001), and cubic fits (GM: AIC = 46133.7, *p* < .0001; WM: AIC = 45354.71, *p* < .0001). Changes across the lifespan and the first 5 years of life for the average GM and WM volumes are depicted in S2–S4 Figs.

In summary, correcting for total brain volume reduced or leveled out the differences in GM and WM between male and female participants. An overall decrease in both partial volumes was found when a lifespan approach was implemented, whereas in the 0–5 age range the increase in GM occurred with a decrease in WM. For all considered measures and age ranges the exponential pattern best described the changes in GM and WM.

### 3.2 Diffusion measures

**Fig 5:**
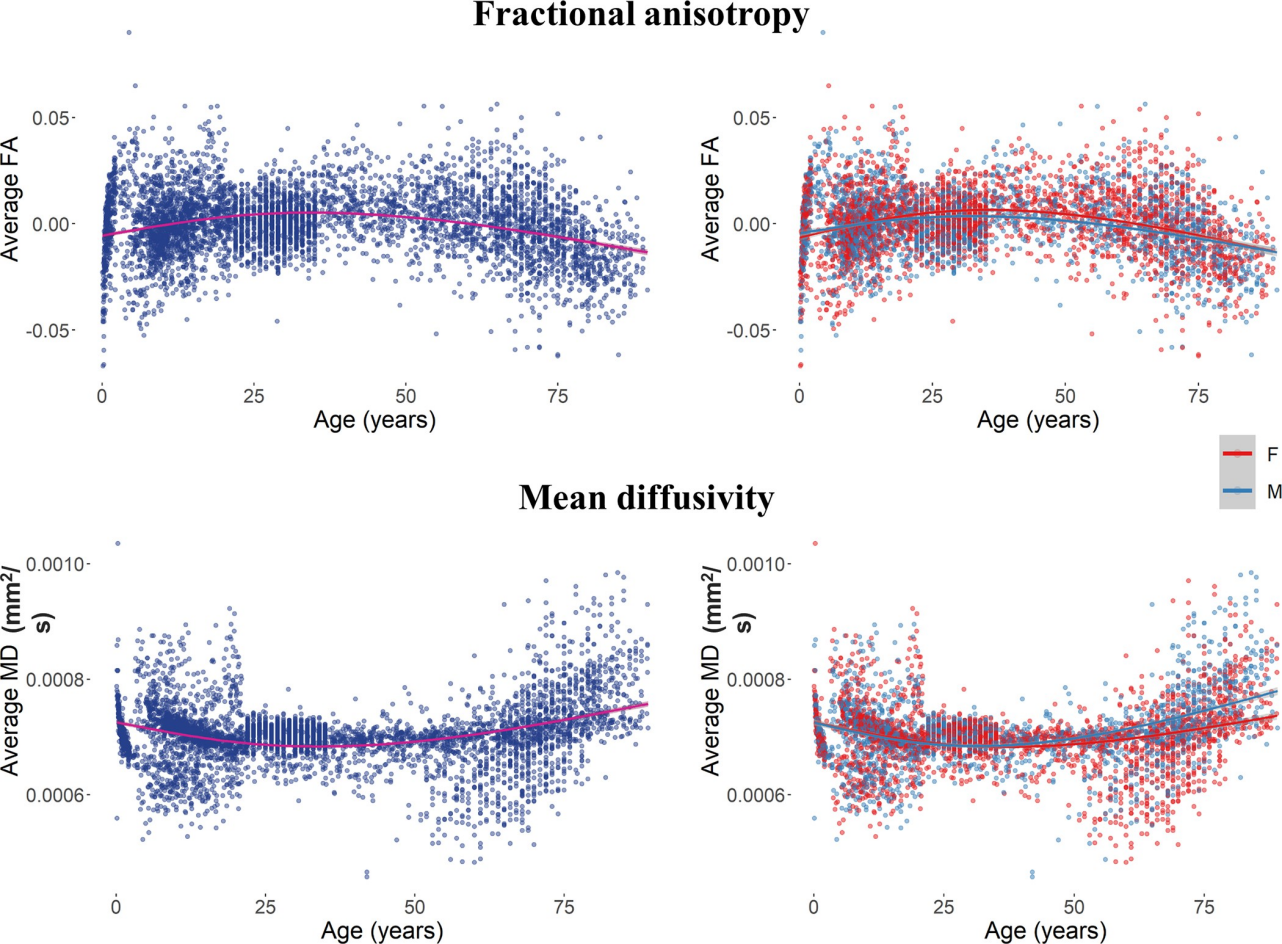
Average FA and MD for the whole sample (left) and as a function of biological sex (right) across the lifespan. Plotted values represent the residuals of a null model for each measure.

There was a change in the average FA values across WM voxels that was unrelated to the effect of biological sex, whereas the MD change occurred at a different rate for male and female participants. Fig 5 depicts the average FA and MD across the lifespan for the whole sample and as a function of the participant’s sex. There was a relation between the change in the FA value and age (beta = -0.0005, *F*(1,5251) = 86.38, *p* < .001), regardless of participant’s biological sex (beta = 0.0013, *F*(1,5251) = 1.62, *p* = .204) and its interaction with age (beta = 0.00003, *F*(1,5251) = 1.48, *p* = .225). On the other hand, the MD values showed to be related to the interaction between age and sex (beta = -0.0000003, *F*(1,5121) = 46.07, *p* < .001), and to the effect of sex (beta = 0.000008, *F*(1,5121) = 13.66, *p* = .002) and age (beta = 0.000002, *F*(1,5121) = 209.94, *p* < .001). These results indicate a decrease in the FA values and an increase in the MD values across the lifespan. The MD changes occur at a different rate between male and female participants, with a larger increase in MD for males at older ages.

We compared the model fits of null, linear, quadratic, cubic, and exponential models to the age change for both average measures. The FA change was best described by the exponential model (AIC = -26259.53; beta = 0.107, *F*(1,5251) = 485.86, *p* < .0001). The exponential model provided a better fit than the cubic (AIC = -25796.55, *p* < .0001), quadratic (AIC = -25616.88, *p* < .0001), and linear (AIC = -24848.24, *p* < .0001) models. Similarly, the best-fit model for the change in MD was the exponential model (AIC = -83804.59; beta = -0.0002, *F*(1,5121) = 331.42, *p* < .0001), which described the data better than the remaining models (cubic model: AIC = -83485.87, *p* < .0001; quadratic model: AIC = -83484.17, *p* < .0001; linear model: AIC = -82491.18, *p* < .0001).

**Fig 6:**
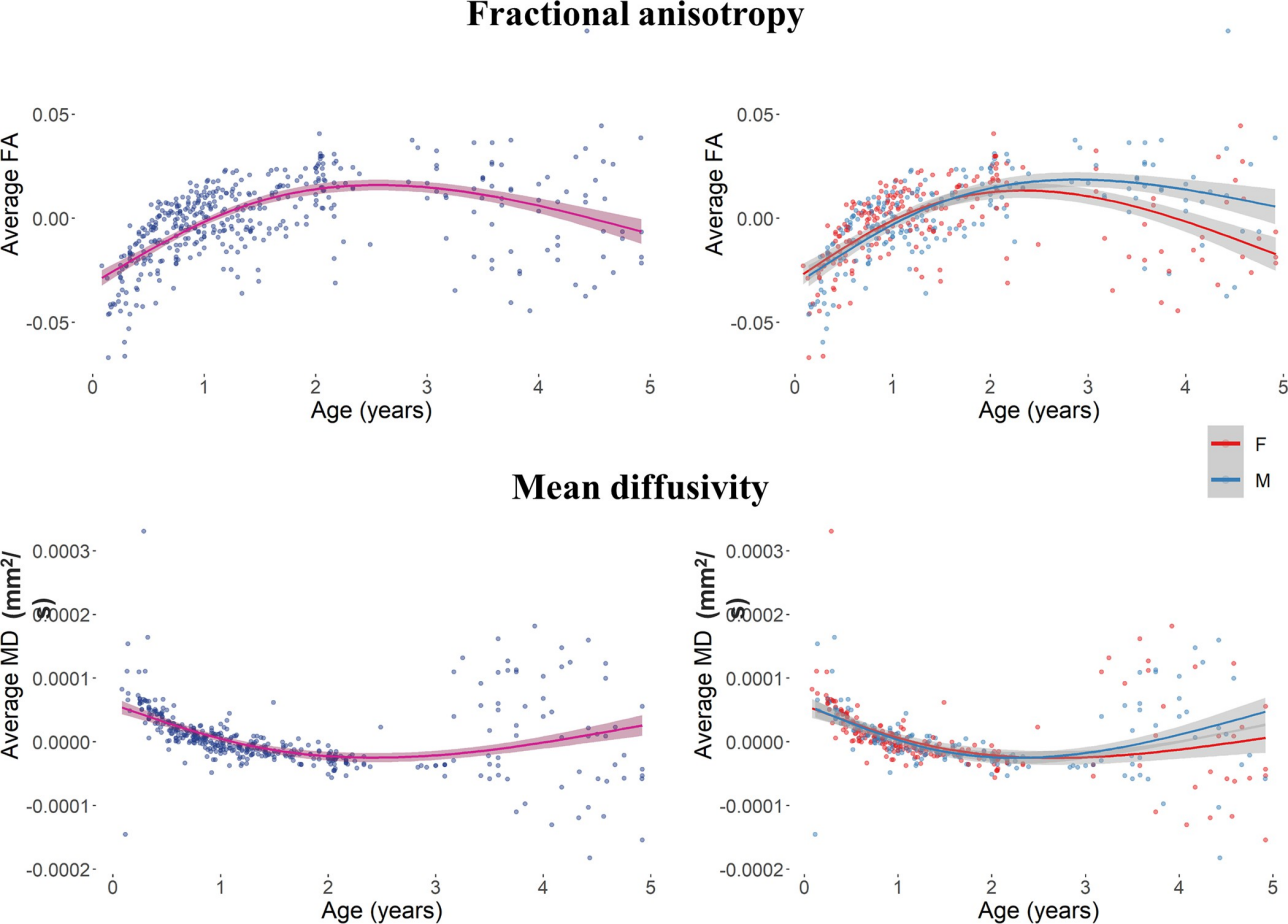
Average FA and MD for the 0–5 age range. The volume changes are plotted for the whole sample (left) and as a function of biological sex (right) across the lifespan.

FA and MD values were analyzed over the first 5 years of life. The distributions of residual values are reported in Fig 6 for the whole sample and as a function of biological sex. Results for the 0–5 age range showed a relation between the FA change and both Age (beta = 0.031, *F*(1,416) = 116.37, *p* < .001) and Sex (beta = 0.006, *F*(1,416) = 10.63, *p* = .007) factors. After an initial similar development, the change in FA for males is larger than the change for females (Fig 5, top right panel). Results for the MD volumes showed a relation to the main effects of Age (beta = -0.00005, *F*(1, 414) = 115.00, *p* < .001), but not to the main effect of Sex (beta = -0.000004, *F*(1, 414) = 0.375, *p* = .541) not to the interaction effect of Age and Sex (beta = 0.000004, *F*(1, 414) = 1.711, *p* = .192).

When tested over the four model fits the FA and MD development over the first five years of life showed that FA was best described by the exponential model (AIC = -1962.49; beta = 0.276, *F*(1,416) = 10.52, *p* = .0013), with a significant improvement than the linear (AIC = -1843.00, *p* < .001), quadratic (AIC = -1925.00, *p* < .001), and cubic fits (AIC = -1953.99, *p* = .001). The best fit for the MD change was described by the exponential model (AIC = -6166.18; beta = -0.0007, *F*(1,416) = 21.27, *p* < .0001). The age-change described by the exponential model was significantly different than the linear model (AIC = -6092.42, *p* < .001) and quadratic (AIC = -6122.39, *p* < .001) models, and cubic model (AIC = -6147.27, *p* < .001).

Overall, these results speak in favor of a decrease in FA and increase in MD across the lifespan. A similar trend occurred when considering the first five years of life only, with an increase in FA and with a lesser degree in MD. Biological sex differences showed an effect on the white matter MD values, which larger in male than female only when the lifespan range was considered. On the other hand, male participants showed larger FA values than female subjects only in the first five years of life, suggesting that early differences in the rate of change of microstructural WM properties are visible when focusing only on young participants. Lastly, all measure were characterized by an exponential change except for the MD change in the 0–5 age range, when the exponential model did not show significant improvement from the cubic model. This result confirms that a more complete picture of the changes that characterize early stages of development can be obtained by including analyses on the first five years of life. It is worth noting that the overall decrease of FA and increase of MD indicated by statistical models may hide more complex patterns of development that are visible from a visual inspection of the data distributions. Our results may inform future studies aiming at identifying specific developmental landmarks for changes in WM volume and its properties [67].

### 3.3 Tractography

**Fig 7:**
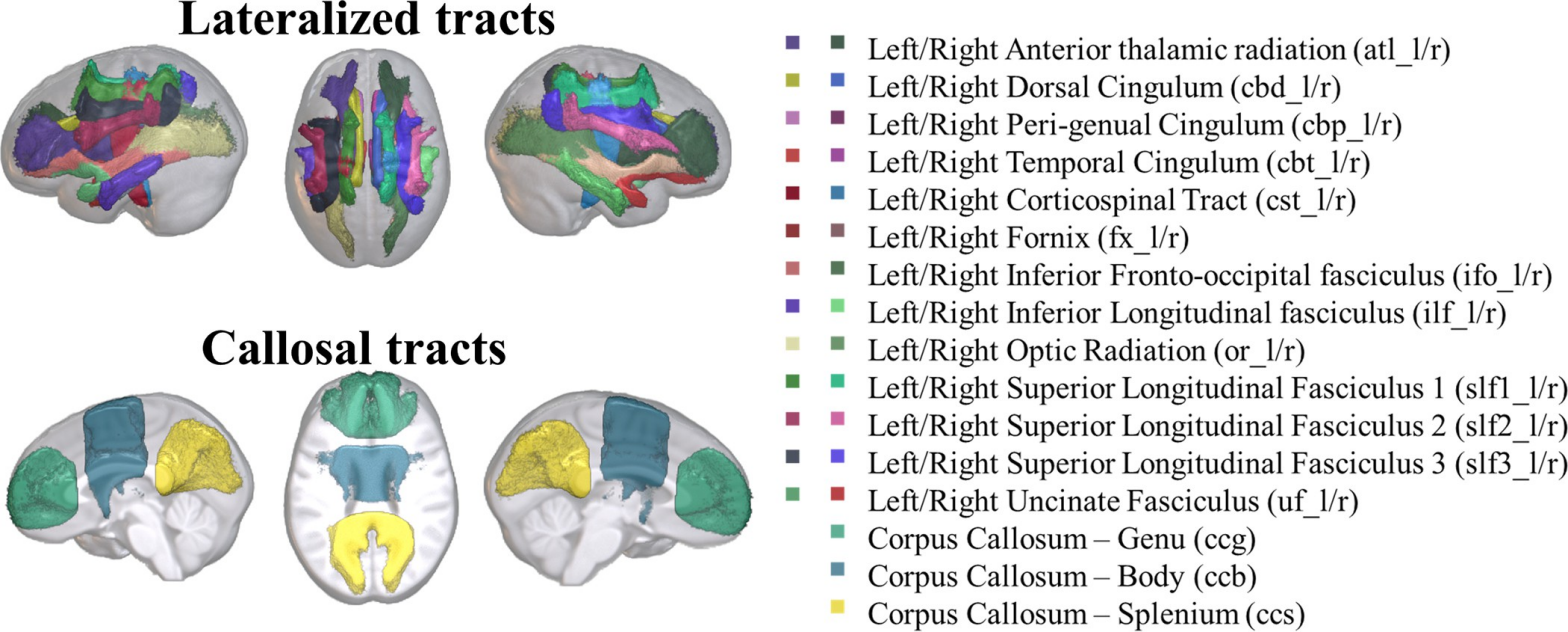
WM tracts defined via probabilistic tractography and displayed on a young-adult template.

In Fig 7 are displayed the reconstructed tract for this study on a young-adult average template. Additional volumes from representative subjects of the infant and adult groups are displayed in S5 Fig included in the supplementary material. We reconstructed 13 tracts per hemisphere and additional three callosal tracts of fiber bundles crossing the two hemispheres.

The FA change was related to the age factor (beta = -0.0004, *F*(1,148579) = 36.69, *p* < .0001) and the interaction between age and sex (beta = 0.00008, *F*(1,148579) = 4.14, *p* = .042). FA values decreased across the lifespan for all tracts, with males showing higher FA values than females. No other factor nor interaction was significantly related to the FA change across tracts.

**Fig 8:**
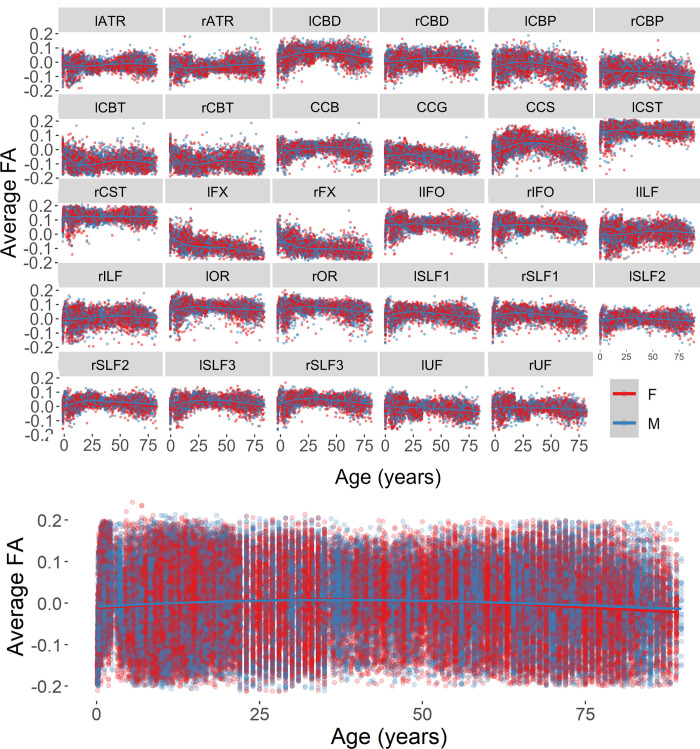
Distributions of residual values for the average FA for each reconstructed tract (top) and across tracts (bottom) as a function of participant’s biological sex.

**Fig 9:**
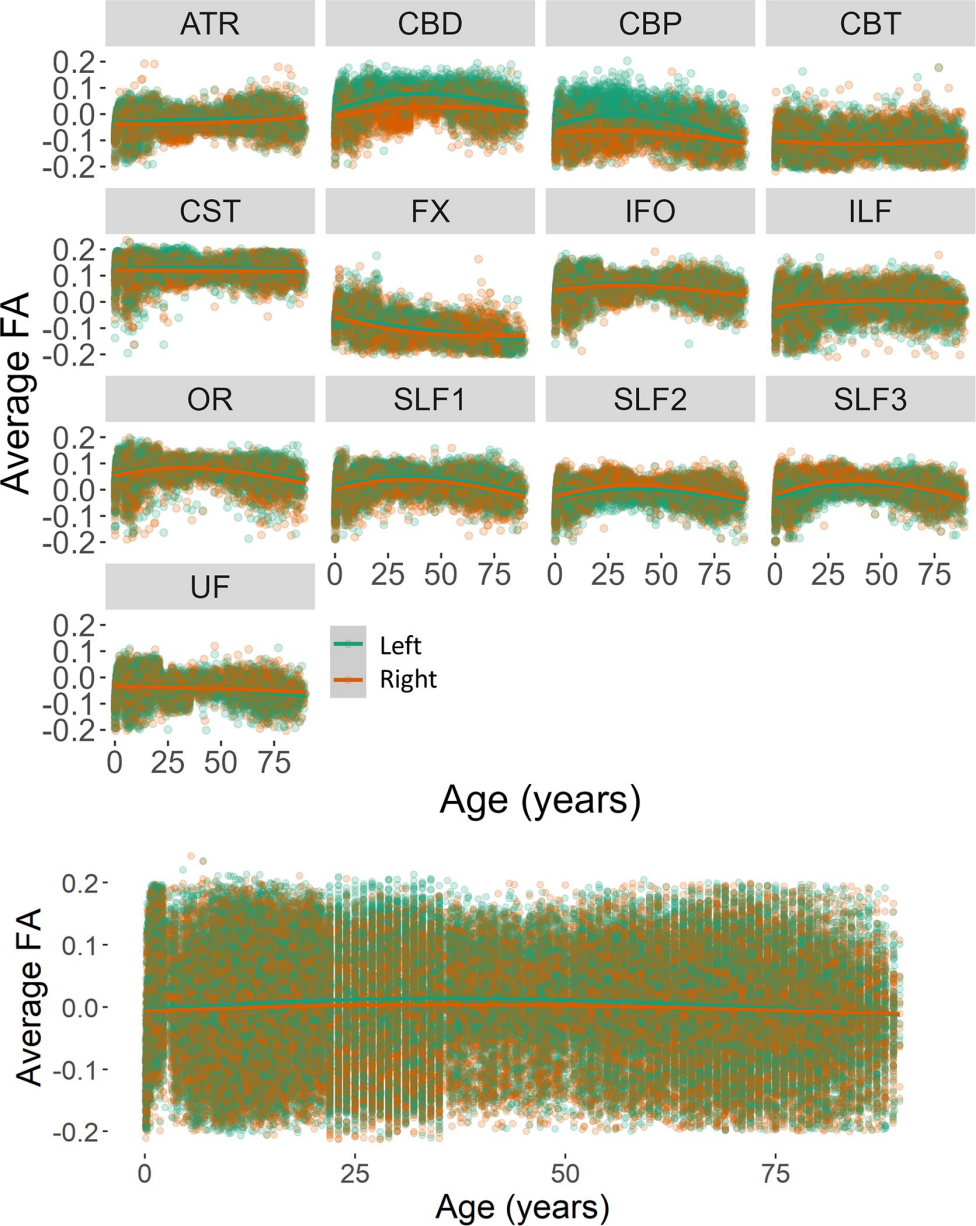
Distributions of residual values for the average FA for each reconstructed tract (top) and across tracts (bottom) as a function of hemisphere.

Hemisphere was added as a fixed factor to the model explaining the change in FA for the lateralized tracts. There was a relation between the FA change and the factors age (beta = -0.0004, *F*(1,132548) = 18.59, *p* < .0001), tract (beta = 0.0015, *F*(1,132548) = 14.26, *p* = .0002), and hemisphere (beta = -0.0835, *F*(1,132548) = 93.86, *p* < .0001). The change in FA values was also related to the interactions between tract and hemisphere (beta = 0.0028, *F*(1,132548) = 25.16, *p* < .0001). Figs 8 and 9 report the FA values across the lifespan as a function of sex and hemisphere separately for each tract and across tracts.

The average FA decreased quicker for female participants (beta = -0.00044) than for male participants (beta = -0.00041). Moreover, the effect of the hemisphere was significant for all tracts except for the uncinate fasciculus, in which the FA change was similar for the left and right hemispheres (Table 2).

**Table 2:** Hemisphere effect results for each lateralized tract under investigation.

Tract	beta	*F*	*p*	Left	Right
ATR	-0.009	1024.9	< .0001	0.4334	0.4241
CBD	-0.039	6342.2	< .0001	0.5116	0.4728
CBP	-0.047	2898.6	< .0001	0.4357	0.3892
CBT	0.0022	14.712	0.0001	0.3457	0.3480
CST	-0.009	392.14	< .0001	0.5899	0.5791
FX	-0.010	232.04	< .0001	0.3759	0.3643
IFO	-0.004	172.97	< .0001	0.5153	0.5105
ILF	0.004	40.666	< .0001	0.4501	0.4538
OR	-0.004	101.19	< .0001	0.5285	0.5247
SLF1	-0.009	448.92	< .0001	0.4856	0.4768
SLF2	0.0114	1102.5	< .0001	0.4455	0.4570
SLF3	0.0106	858.15	< .0001	0.4592	0.4697
UF	0.0005	1.6399	0.200	0.4159	0.4165

Null, linear, quadratic, cubic, and exponential models to the age change in FA values were fitted for all 29 tracts. The age change in FA for all tracts was better described by the exponential model (all *p*s < .001). The corresponding AIC values for each tract and model fit are reported in Table 3.

**Table 3:** AIC values of each model fit for the FA analyses on all considered tracts.

Tract	AIC null		AIC linear		AIC quadratic		AIC cubic		AIC exponential	
	Left	Right	Left	Right	Left	Right	Left	Right	Left	Right
ATR	-19084	-19168	-19120	-19249	-19190	-19454	-19198	-19544	-19402	-19964
CBD	-17543	-21164	-17547	-21193	-17596	-21483	-17637	-21607	-17764	-21914
CBP	-14677	-18731	-14909	-18775	-15034	-19128	-15039	-19262	-15196	-19609
CBT	-19044	-15940	-19130	-16055	-19527	-16213	-19672	-16254	-20145	-16441
CCB	-20798.38 (not lateralized)		-20830.36 (not lateralized)		-21191.78 (not lateralized)		-21314.82 (not lateralized)		-21620.78 (not lateralized)	
CCG	-18154.48 (not lateralized)		-18190.40 (not lateralized)		-18603.66 (not lateralized)		-18739.98 (not lateralized)		-19120.04 (not lateralized)	
CCS	-14358.21 (not lateralized)		-14412.59 (not lateralized)		-14681.08 (not lateralized)		-14729.06 (not lateralized)		-14884.40 (not lateralized)	
CST	-16940	-20583	-16995	-20648	-17105	-20924	-17121	-21095	-17337	-21787
FX	-19168	-20006	-19197	-20073	-19514	-20486	-19670	-20714	-20016	-21461
IFO	-19528	-19651	-19638	-19759	-20081	-19977	-20228	-20050	-20696	-20481
ILF	-19132	-18326	-19195	-18337	-19668	-18402	-19798	-18405	-20491	-18562
OR	-20581	-19281	-20648	-19289	-20918	-19427	-21044	-19502	-21809	-19659
SLF1	-17545	-20255	-17594	-20378	-17755	-20920	-17785	-21017	-18104	-21364
SLF2	-18860	-20508	-18903	-20642	-19307	-20934	-19456	-21046	-19745	-21607
SLF3	-18882	-16491	-18949	-16551	-19339	-17134	-19440	-17228	-20050	-17461
UF	-19811	-15367	-19888	-15478	-20223	-15536	-20373	-15550	-21055	-15690

The same analytical strategy was applied to the investigation of MD changes in the selected tracts. We found a significant interaction between tract and sex (beta = 0.0000003, *F*(1,24793) = 9.06, *p* = .003). MD values for males and females changed at a different rate for the various tracts and showed larger MD values in female (*M* = 0.00063, *SE* = 0.000002) rather than the male (*M* = 0.00060, *SE* = 0.000001) participants. No other factor nor interactions were significantly related to the MD change across tracts. There was not a significant age change in MD for the considered tracts.

**Fig 10:**
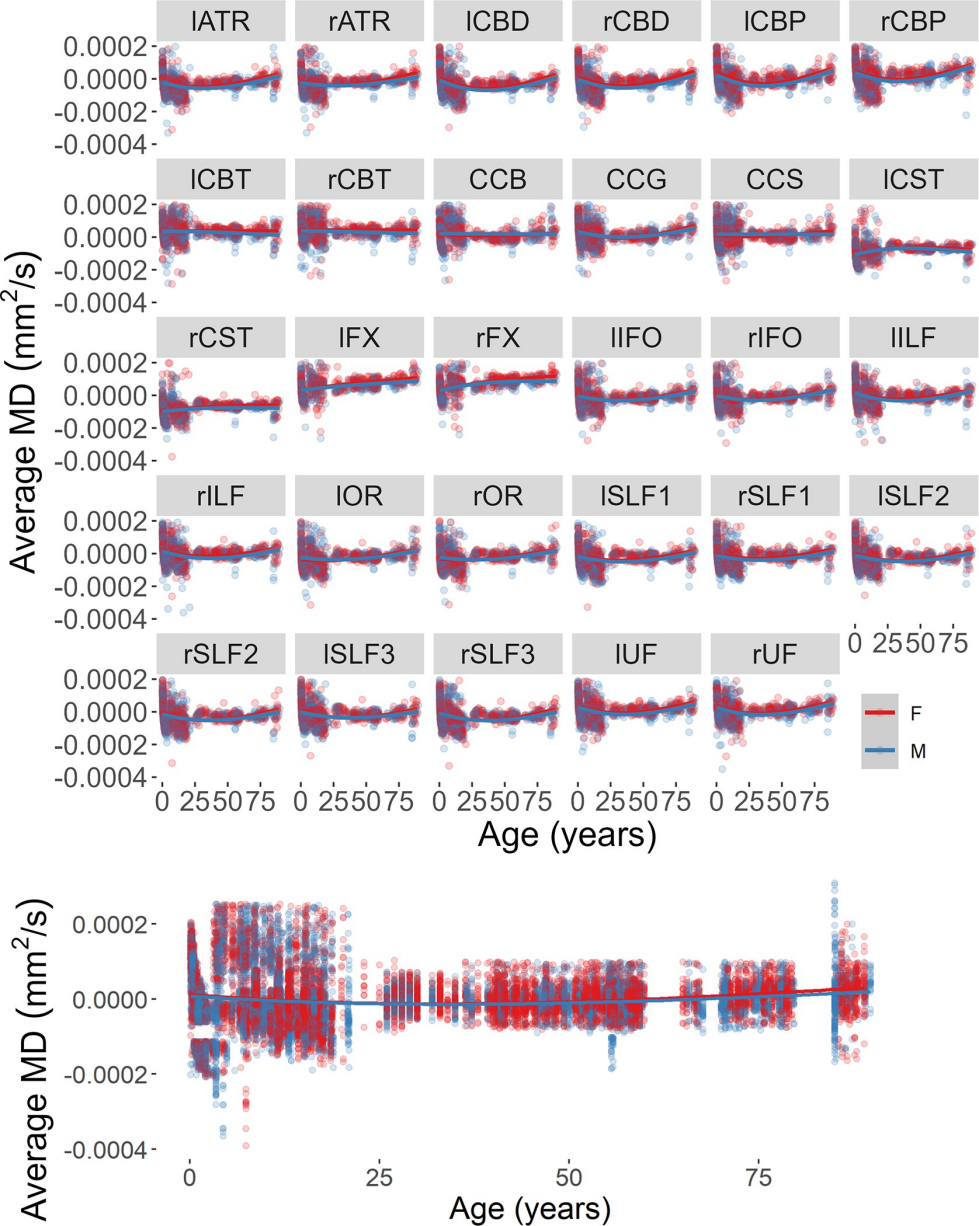
Average MD for each reconstructed tract (top) and across tracts (bottom) as a function of participant’s biological sex.

**Fig 11:**
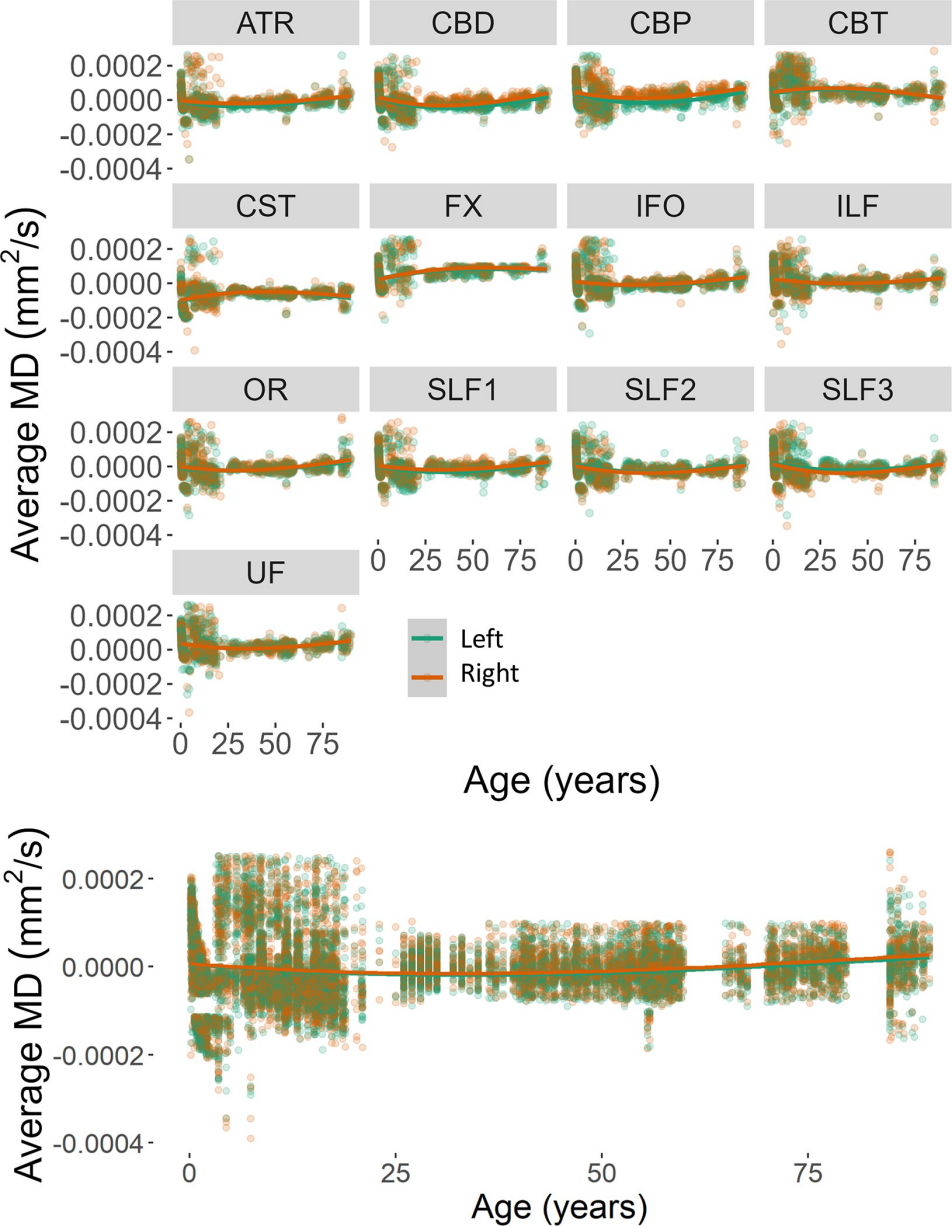
Average MD for each reconstructed tract (top) and across tracts (bottom) as a function of hemisphere.

There were no significant main effects nor interactions when the Hemisphere was added as a fixed factor to the model explaining the change in MD for the lateralized tracts (*p*s > 0.06). Figs 10 and 11 report the distribution of residual values for the MD measure across the lifespan as a function of sex and hemisphere separately for each tract and across tracts.

Null, linear, quadratic, cubic, and exponential models to the age change in MD values were fitted for all 29 tracts. The age change in MD for all tracts was better described by the exponential model (all *p*s < .001). The corresponding AIC values for each tract and model-fit are reported in Table 4.

**Table 4:** AIC values of each model fit for the MD analyses on all considered tracts.

Tract	AIC null		AIC linear		AIC quadratic		AIC cubic		AIC exponential	
	Left	Right	Left	Right	Left	Right	Left	Right	Left	Right
ATR	-14685	-14903	-14683	-14903	-14799	-15032	-14815	-15050	-15092	-15401
CBD	-13885	-14973	-13883	-14972	-14030	-15049	-14052	-15067	-14342	-15297
CBP	-13956	-15426	-13956	-15424	-14014	-15571	-14020	-15595	-14274	-15975
CBT	-14332	-14474	-14331	-14472	-14415	-14629	-14420	-14659	-14574	-15060
CCB	-14629.60 (not lateralized)		-14627.87 (not lateralized)		-14750.97 (not lateralized)		-14771.96 (not lateralized)		-14977.95 (not lateralized)	
CCG	-14843.95 (not lateralized)		-14842.29 (not lateralized)		-14972.14 (not lateralized)		-15004.88 (not lateralized)		-15496.59 (not lateralized)	
CCS	-14870.36 (not lateralized)		-14868.91 (not lateralized)		-14932.39 (not lateralized)		-14934.48 (not lateralized)		-15137.15 (not lateralized)	
CST	-10848	-14257	-11170	-14256	-10879	-14388	-10847	-14418	-10894	-14661
FX	-14630	-14605	-14628	-14603	-14751	-14730	-14772	-14755	-14978	-15240
IFO	-15021	-14714	-15021	-14713	-15238	-14875	-15278	-14907	-15652	-15198
ILF	-14332	-14190	-14331	-14188	-14415	-14300	-14420	-14328	-14574	-14737
OR	-15605	-13973	-15606	-13972	-15734	-14154	-15767	-14182	-16080	-14401
SLF1	-14844	-14860	-14842	-14859	-14972	-14951	-15005	-14963	-15497	-15197
SLF2	-9492.8	-14342	-9490.8	-14340	-9530.8	-14447	-9545.9	-14477	-9819.2	-14727
SLF3	-15244	-14203	-15242	-14201	-15389	-14324	-15420	-14353	-15642	-14690
UF	-14721	-15315	-14719	-15314	-14849	-15401	-14874	-15419	-15241	-15801

Density values were analyzed as a function of the participant’s age, biological sex, white matter tract, and interaction factors. Changes in density were related to the age (beta = 0.00002, *F*(1,139005) = 27.41, *p* < .0001) and tract (beta = -0.00008, *F*(1,139005) = 111.09, *p* < .0001) factors, as well as their interaction (beta = -0.000002, *F*(1,139005) = 190.76, *p* < .0001). Age-related changes in the variation of tract density were investigated separately for each considered tract. Results are summarized in Table 5. The inferior fronto-occipital fasciculi, superior longitudinal fasciculi (II), and the splenium of the corpus callosum did not show a significant change in their density values across ages. Bilateral changes occurred for the temporal cingula, inferior longitudinal fasciculi, superior longitudinal fasciculi (sections I and III), uncinate fasciculi, and optic radiations. Density changes occurred on the left hemisphere for the anterior thalamic radiation, dorsal cingulum, peri-genual cingulum, and fornix, with a density decrease over age for dorsal cingulum and fornix tracts and an increase for the remaining tracts. The right cortico-spinal tract and the body of the corpus callosum showed to significantly decrease across ages, whereas density increased in the genu of the corpus callosum.

**Table 5:** Summary of the results on the age-related differences in tract density.

Tract	*b*	*F*	*p*
ATR Left *	0.000100	22.3965	< .0001
ATR Right	-0.000069	1.63601	0.200933
CBD Left *	-0.000194	5.51553	0.018927
CBD Right	0.000074	1.91457	0.166518
CBP Left *	0.000097	5.74502	0.016575
CBP Right	-0.000001	7.3E-05	0.993196
CBT Left *	0.000089	6.4388	0.011196
CBT Right *	-0.000262	52.1363	< .0001
CST Left	0.000023	1.00649	0.315792
CST Right *	-0.000223	25.8972	< .0001
FX Left *	-0.000136	9.88289	0.001678
FX Right	0.000003	0.01016	0.919711
ILF Left *	-0.000115	8.89996	0.002865
ILF Right *	0.000069	13.0373	0.000308
IFO Left	-0.000047	1.33146	0.248601
IFO Right	-0.000080	1.0893	0.296722
SLF1 Left *	0.000149	7.27857	0.007002
SLF1 Right *	0.000107	7.71946	0.005484
SLF2 Left	-0.000104	2.57202	0.108878
SLF2 Right	0.000053	2.5538	0.110091
SLF3 Left *	-0.000274	69.1339	< .0001
SLF3 Right *	0.000081	10.0997	0.001491
UF Left *	-0.000150	10.7622	0.001043
UF Right *	-0.000078	4.23448	0.039662
OR Left *	-0.000104	11.9296	0.000557
OR Right *	-0.000193	23.5206	< .0001
CCB *	-0.000003	13.2637	0.000273
CCG *	0.000010	50.9419	< .0001
CCS	-0.000001	0.17307	0.677414

**Fig 12:**
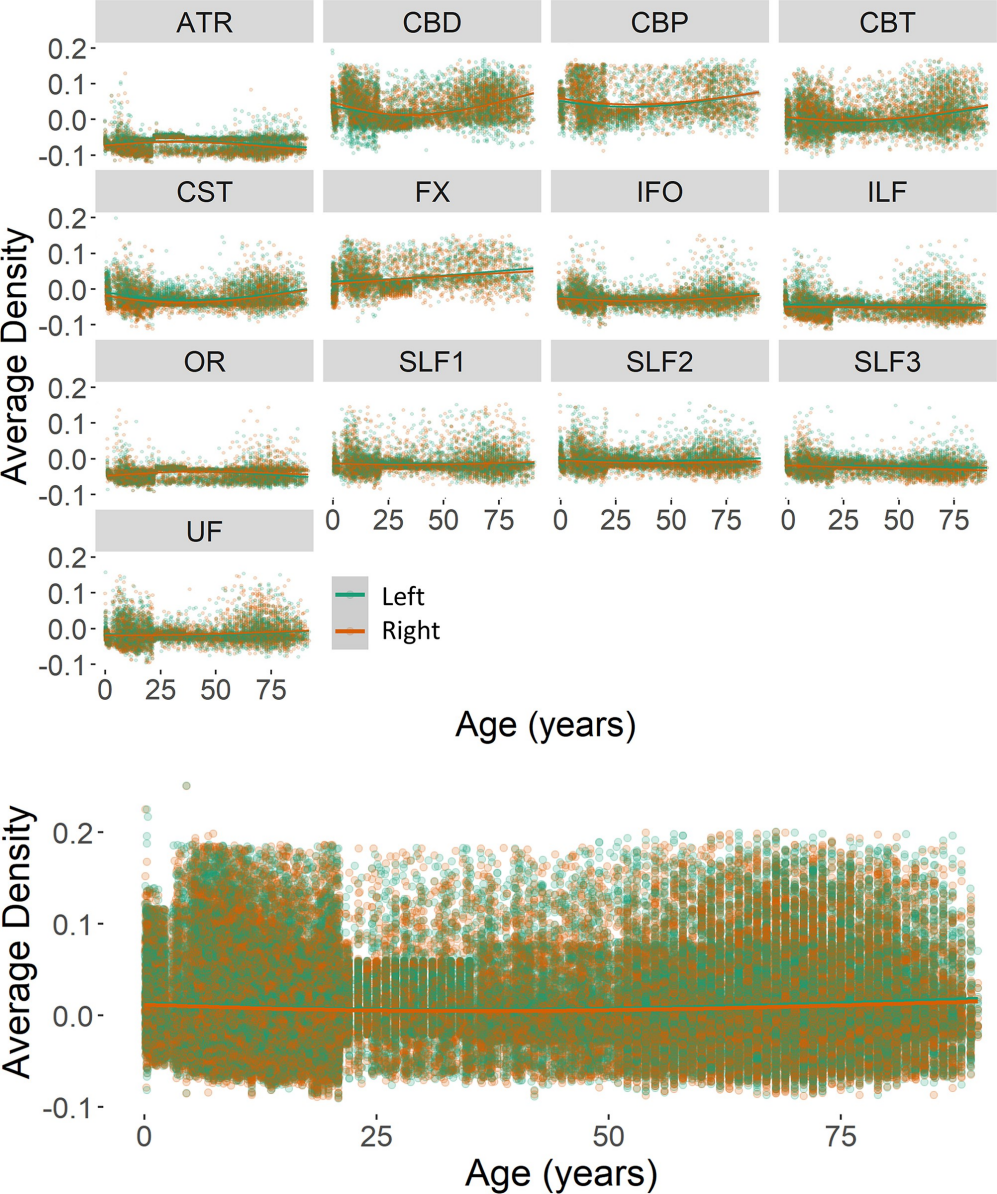
WM density values for each lateralized tract (top) and across tracts (bottom) as a function of hemisphere (right panels). Plotted values represent the residuals of the null model.

When hemisphere was included as a factor for the lateralized tracts, the density change showed to significantly be related to tract type (beta = 0.0002, *F*(1, 122881) = 17.84, *p* < .0001) and its interaction with hemisphere (beta = -0.00007, *F*(1, 122881) = 4.016, *p* = .045). Overall, greater density values characterized the tracts on the left (*M* = .079 *SE* = .0002) rather than right (*M* = .077 *SE* = .0002) hemisphere (Fig 12).

Null, linear, quadratic, cubic, and exponential models were fitted for all 29 tracts to define the age change in tract density. A cubic change showed to describe the density values across ages for the body of the corpus callosum, bilateral anterior thalamic radiation, dorsal cingulum, and left superior longitudinal fasciculus and fornix. For all remaining tracts, the best model fit was the exponential model. The corresponding AIC values for each tract and model fit are reported in Table 6.

**Table 6:** AIC values of each model fit for the analyses on the density values of all considered tracts.

Tract	AIC null		AIC linear		AIC quadratic		AIC cubic		AIC exponential	
	Left	Right	Left	Right	Left	Right	Left	Right	Left	Right
ATR	-28770	-6481.7	-28772.3	-6486.4	-28790	-6484.5	-28821.6	-6489.3	-28821	-6487.3
CBD	-20511	-34670	-20510.33	-34676	-20509	-34689	-20518.2	-34694	-20516	-34692
CBP	-79825	-30149	-79857.71	-30154	-80008	-30249	-80043.8	-30359	-80060	-30386
CBT	-26271	-28440	-26273.12	-28442	-26281	-28469	-26287.7	-28483	-26297	-28483
CCB	-49752.84 (non lateralized)		-49755.8 (non lateralized)		-49804.82 (non lateralized)		-49815.94 (non lateralized)		-49814.85 (non lateralized)	
CCG	-29705.81 (non lateralized)		-29720.78 (non lateralized)		-29767.16 (non lateralized)		-29831.16 (non lateralized)		-29857.38 (non lateralized)	
CCS	-4458.11 (non lateralized)		-4462.712 (non lateralized)		-4461.502 (non lateralized)		-4462.217 (non lateralized)		-4464.566 (non lateralized)	
CST	-29528	-29935	-29526.89	-29947	-29552	-29954	-29570.1	-29980	-29579	-29984
FX	-35324	-28372	-35331.45	-28374	-35341	-28374	-35380.6	-28400	-35394	-28398
IFO	-29688	-28717	-29696.2	-28734	-29724	-28765	-29772.9	-28769	-29785	-28781
ILF	-31010	-33112	-31008.07	-33138	-31012	-33143	-31040.3	-33188	-31042	-33191
OR	-50382	-36174	-50380.39	-36173	-50409	-36182	-50414.6	-36186	-50418	-36186
SLF1	-76314	-26249	-76312.19	-26247	-76465	-26259	-76487.8	-26271	-76513	-26275
SLF2	-24985	-82561	-24984.91	-82568	-25002	-82657	-25012.8	-82657	-25027	-82722
SLF3	-30313	-13257	-30325.78	-13258	-30388	-13266	-30496.8	-13271	-30522	-13270
UF	-8327.6	-26100	-8335.583	-26100	-8342.8	-26116	-8377.8	-26122	-8426.1	-26142

Overall, an exponential change occurred for most tracts and considered measures (i.e., FA, MD, and density). Only density changes in the dorsal and peri-genual cingula (left hemisphere), and the uncinate fasciculus, dorsal cingulum, and fornix (right hemisphere) were better described by the cubic fit. These results confirm that model fit outputs for most of the individual tracts mirror the nonlinear fits obtained across the brain, and suggest a differential fit (i.e., cubic) for density measures in tracts of the cingulate cortex (i.e., left and right CBD and left CBP), the right fornix and uncinate fasciculus. Changes in MD showed to be related to the sex factor with a larger increase in MD across all tracts in female than male participants. Similarly, females showed a greater decrease in FA than males, regardless of the considered tract. Hemispheric asymmetries occurred in both FA and tract density for all tracts except for the uncinate fasciculus. Both measures showed increased leftward asymmetry irrespective of the tract under investigation. Further studies are needed to investigate whether such asymmetry is driven by or linked to functional brain responses.

## 4. Discussion

In the current study, we investigated the lifespan change in WM partial volume and its properties by combining structural MRI and DWI volumes from several datasets of subjects 0–100 years. The availability a dataset that covers a wide age range allowed us to investigate the trajectories of brain development across the lifespan and to extend the current knowledge coming from studies focusing on each developmental stage separately. We tested different curve fits to assess which model best explained the age changes in WM and assessed individual differences in terms of biological sex and hemispheric pattern of development and aging. These changes were investigated both brain-wide and for 16 major associative and callosal WM fiber bundles reconstructed through probabilistic tractography.

Results on the total volume change showed sex differences between male and female participants only on uncorrected GM and WM volumes. When correcting for participants’ brain volume size, the overall sex differences were nonsignificant. However, the interaction between participants’ age and biological sex suggested that the effect of sex varies across the lifespan. Specifically, it seems that GM changes are greater for females than males, whereas WM changes follow the opposite pattern starting in young adulthood. When considering the first five years of life only, the development of GM and WM partial volumes occurs at the same rate for female and male participants.

These results are in line with previous evidence suggesting greater integrity of WM in male than female youths and greater GM decrease in males than females [68]. Biological sex differences early in life are reported to be limited to the caudate and cerebellar vermis when considering normalized brain volumes [69]. It is possible that hormonal changes occurring during puberty contribute to the differential trajectories in GM and WM between males and females. This investigation was behind the scope of the current work and should be further examined in future studies. There is evidence of sex-related differences in both cortical and subcortical development [70] that is not always replicated [71]. Several confounding factors (e.g., sampling strategy, acquisition protocols) and individual variabilities, which have been found to be greater in boys [72], may be responsible for the variations in findings between studies.

Lifespan changes in brain development and aging can be investigated in terms of total and partial brain volumes and in relation to the microstructural properties of GM and WM, although the most significant changes in these metrics have been explored almost exclusively for WM. Here, we tested whether sex-related differences interact with participants’ age to explain the changes of two major WM properties, i.e., FA and MD, across the lifespan. Results suggest that both brain-wide WM metrics show a change across the lifespan, with an overall increase in FA and decrease in MD. This latter metric also showed differential trajectories between males and females, with larger MD values reported for boys than girls, starting from the second half of the age period considered in the current study.

FA and MD refer to complementary properties of water diffusion: high values of FA are indicative of a preferential diffusion along one main direction (i.e., anisotropic diffusion), whereas high MD values are indicative of isotropic diffusion [73]. Despite their complementary nature, the reciprocal development of FA and MD does not seem to follow an inverse relationship. This may be partially due to the limited ability of tensor-based methods (i.e., DTI) in accurately modeling the anisotropy of crossing fibers, thus overestimating MD values. Our results suggest that sex-related differences occur for MD but not for FA. MD values in the whole brain are similar for females and males in the first years of life, whereas MD shows a larger increase for boys than girls in adulthood and late adulthood. There is mixing evidence for age-related changes in FA; cross-sectional studies report either no differences [74] or higher FA in females than males in adolescence [75], whereas a longitudinal study showed sex-related differences in childhood and early adulthood, but not during adolescence [76]. Although MD changes are less investigated, Hsu and colleagues (2008) showed a greater MD increase in males than females between 30 and 80 years of age [77].

When considering only young participants, both FA and MD followed the same changing rate between sexes during the first years of life and started to diverge at about 2 years for FA and 3 years for MD, with boys showing higher values in both metrics than girls. These results speak in favor of a differential pattern of development of FA and MD for the two sexes, in which boys seem to show bigger changes than girls. To the best of our knowledge, no study explored sex-related differences in brain-wide WM properties in typically developing infants and children. The very few studies with young pediatric samples reported an overall increase in FA and a decrease in MD [23]. It is important to note that in our study the number of available DWI volumes between 2 and 5 years of life was lower than the available sample at younger ages. This unbalance may be due to the difficulties in acquiring imaging volumes with children between 1 and 3 years of age [78] an age in which there is a higher number of data loss and greater variability in the acquisition protocols (i.e., asleep vs awake acquisitions). The investigation of typical developmental trajectories of FA and MD in young children would benefit from further studies focusing on brain-based sex differences since several neurodevelopmental disorders show different phenotypic profiles in boys and girls [7].

A similar picture is depicted when considering lifespan FA and MD changes for the major WM tracts we reconstructed through probabilistic tractography. Specifically, sex- and age-related changes occurred across all tracts and were only marginally significant for FA (*p =* .042) with a larger decrease for females only in late adulthood. MD values for males and females changed at a different rate for the various tracts under investigation, but none of these effects were also related to the participant’s age. Hemispherical differences in associative tracts were significant only for the FA metric. All tracts except for the uncinate fasciculus showed a lateralization effect, the direction of which varied as a function of the considered tract. Larger FA values were found in the right vs. left tracts for the superior longitudinal fasciculi (sections 2 and 3), inferior longitudinal fasciculus, and temporal cingulum. The strongest left lateralization was evident for the dorsal and the peri-genual cingula (CBD and CBP) but also occurred for all remaining tracts. Most of these differences may be due to the development of functional cognitive processes, however, they should be interpreted with caution given their small magnitude. Two limbic fiber bundles showed the biggest lateralization effect, i.e., CBD and CBP, with larger FA changes for the left hemisphere. These two fibers are part of the more complex cingulum bundle, a WM tract that runs through frontal, parietal, and medial temporal sites and also links subcortical nuclei to the cingulate gyrus. Our results are in line with previous evidence of leftward FA asymmetry in the cingulum bundle in adults [79–81] and extend this evidence to younger ages. Further studies are needed to investigate the role of tract asymmetry in the development of cognitive functions. For instance, the left-over-right FA values have been linked to attention orienting processes in young adults [82] and may benefit from a lifespan approach to clarify the development of this structural-functional relationship.

The last goal of the current study was to model different curve fits to explain the age changes in WM volume and its microstructural properties. Results suggested that age changes in WM and GM volumes are exponential both when considering the lifespan and 0–5 age ranges. The exponential increase also characterized the development of the FA and MD metrics of WM, regardless of the age range considered. Most individual studies applied linear fits over narrowed age ranges and/or variable sample sizes (for a review, see [27]). However, nonlinear changes are reported when wider age ranges are considered. More studies are needed to draw definitive conclusions on these brain changes. The changes occurring on individual tracts showed similar patterns for both FA and MD values, indicating an exponential change in FA and a quadratic trend for MD.

It is worth highlighting that our results come from the use of specific algorithms to estimate and reconstruct WM fiber bundles, and that different approaches to deal with crossing fibers may lead to different conclusions [83]. Similarly, our results are informative on the patterns of WM development and its properties across the entire lifespan. However, more complex patterns may emerge from curve fitting models that breakdown the changes in smaller age ranges. This question may be better addressed from the use of longitudinal datasets with more equal volume numbers across ages. Lastly, additional knowledge on the patterns of WM development may be gained from implementing algorithms that consider the complexity of WM microstructural properties that are neglected by diffusion tensor algorithms [48]. Overall, our results suggested that different patterns of change occur for the WM metrics under investigation. All sex-related differences seemed to be leveled out when WM partial volumes were corrected for the participant’s volume size, although the rate of change differed between males and females in late adulthood. Microstructural properties of WM showed a general increase in FA and decrease in MD, with sex-related differences only in MD. Hemispheric asymmetries in FA occurred in all reconstructed tracts, except for the uncinate fasciculus, whereas changes in MD values did not differ between left and right hemispheres. The exponential fit was the best curve model to explain the WM changes in partial volume, FA (both brain-wide and at the individual tract levels), and brain-wide MD. A quadratic trend was instead the best fit for the MD changes occurring in infancy and childhood and the lifespan changes of each individual tract. These results suggest that specific trajectories may be identified when the whole lifespan is considered over investigations focusing on individual developmental stages. WM changes occur throughout the lifespan following nonlinear pathways that should be further investigated in relation to the functional changes that characterize the various domains of cognition [84].

## Supporting information

S1 Fig FA values as a function if Database plotted as raw values (top) and residuals of modeling Database as RF (bottom).
(PDF)

S2 Fig Residual scores for the changes in GM and WM cross the lifespan (left panels) and the first 5 years of life (right panels).
(PDF)

S3 Fig Panel a) Residual scores for the changes in WM across the lifespan for each lobar region (top left) and their relative change (top right). Heatmap of the total WM volume change for each age group and lobar region (bottom). Panel b) Residual scores of the WM changes (left) and heatmap of WM volume changes (right) for each lobar region for the first five years of life.
(PDF)

S4 Fig Residual scores for the changes in WM partial volume cross the lifespan as a function of the database (left panel). Relative change in WM partial volume across all age groups (right panel).
(PDF)

S5 Fig Axial view of the reconstructed WM tracts from representative infant and adult subjects, along with their sagittal views overlayed to the participant’s T1 scan. (PDF)

S1 Table Total number of volumes for each database. (PDF)

S2 Table Variance and AIC values of each considered brain measure for models with and without database as random effect (RE). (PDF)
