# Supplementary material for: White matter trajectories over the lifespan
Source: PLoS One. 2024 May 17;19(5):e0301520. doi: 10.1371/journal.pone.0301520 (PMC11101104; doi:10.1371/journal.pone.0301520)

Supplementary Figure S1. FA values as a function if Database plotted as raw values (top) and residuals of modeling Database as RF (bottom).

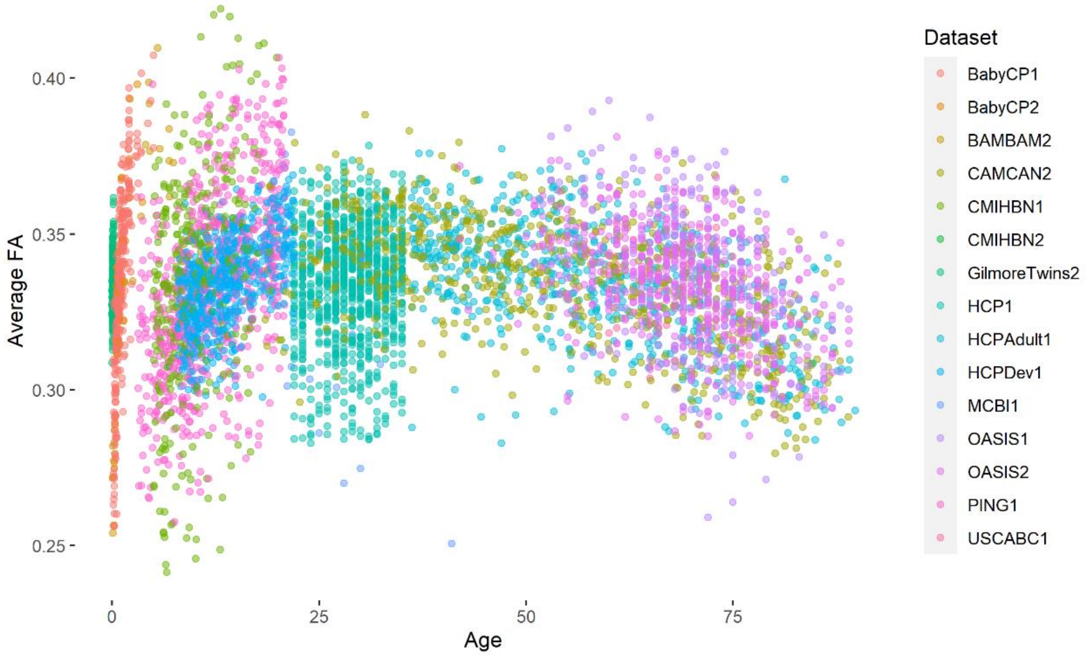

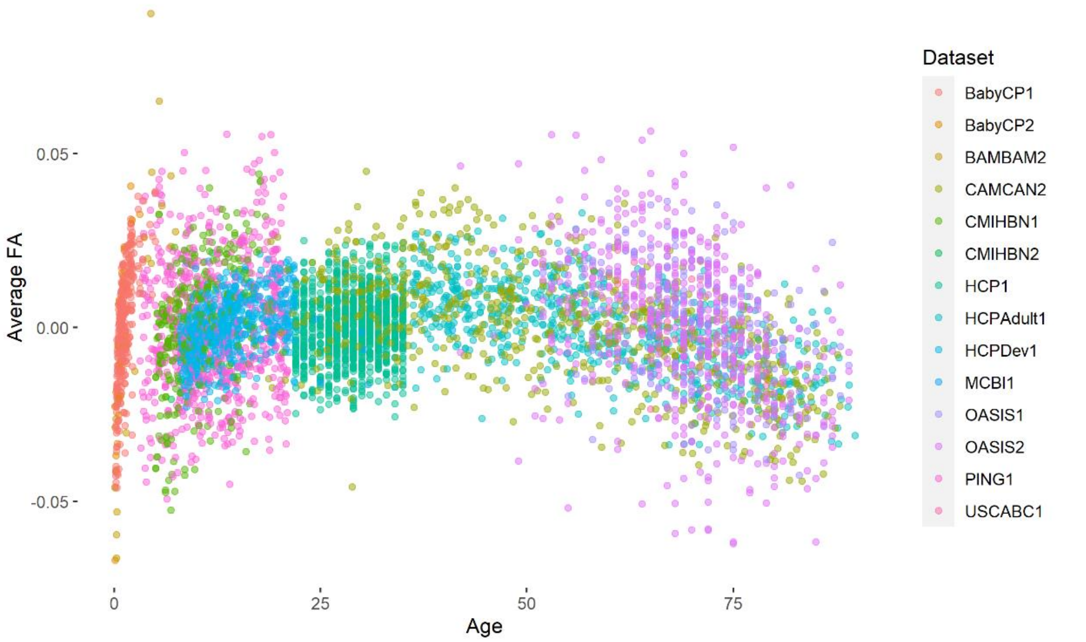

Supplement: S1 Fig — FA values as a function if Database plotted as raw values (top) and residuals of modeling Database as RF (bottom). (PDF) [file pone.0301520.s001.pdf]
