# Supplementary material for: White matter trajectories over the lifespan
Source: PLoS One. 2024 May 17;19(5):e0301520. doi: 10.1371/journal.pone.0301520 (PMC11101104; doi:10.1371/journal.pone.0301520)

Supplementary Figure S2. Residual scores for the changes in GM and WM cross the lifespan (left panels) and the first 5 years of life (right panels).

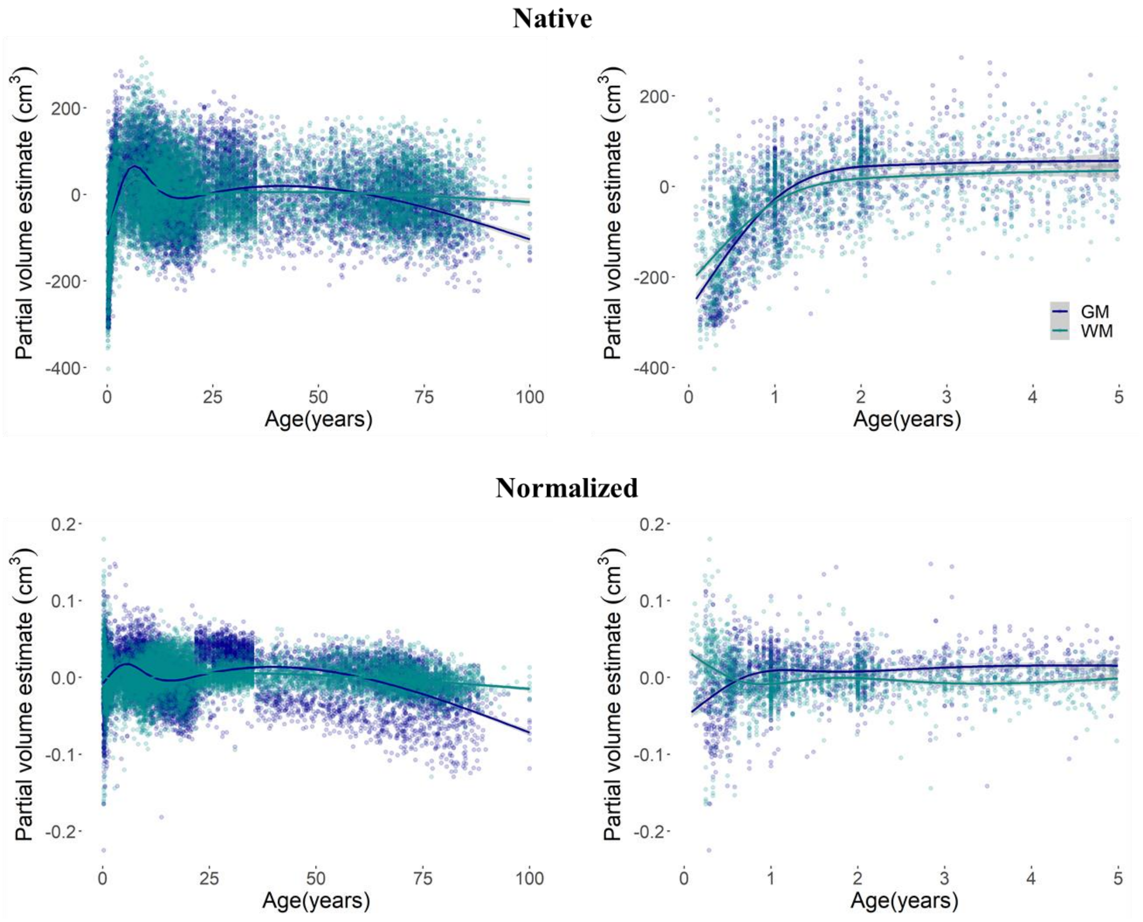

Supplement: S2 Fig — Residual scores for the changes in GM and WM cross the lifespan (left panels) and the first 5 years of life (right panels). (PDF) [file pone.0301520.s002.pdf]
