# Supplementary material for: White matter trajectories over the lifespan
Source: PLoS One. 2024 May 17;19(5):e0301520. doi: 10.1371/journal.pone.0301520 (PMC11101104; doi:10.1371/journal.pone.0301520)

Supplementary Figure S3. Panel a) Residual scores for the changes in WM across the lifespan for each lobar region (top left) and their relative change (top right). Heatmap of the total WM volume change for each age group and lobar region (bottom). Panel b) Residual scores of the WM changes (left) and heatmap of WM volume changes (right) for each lobar region for the first five years of life.

a)

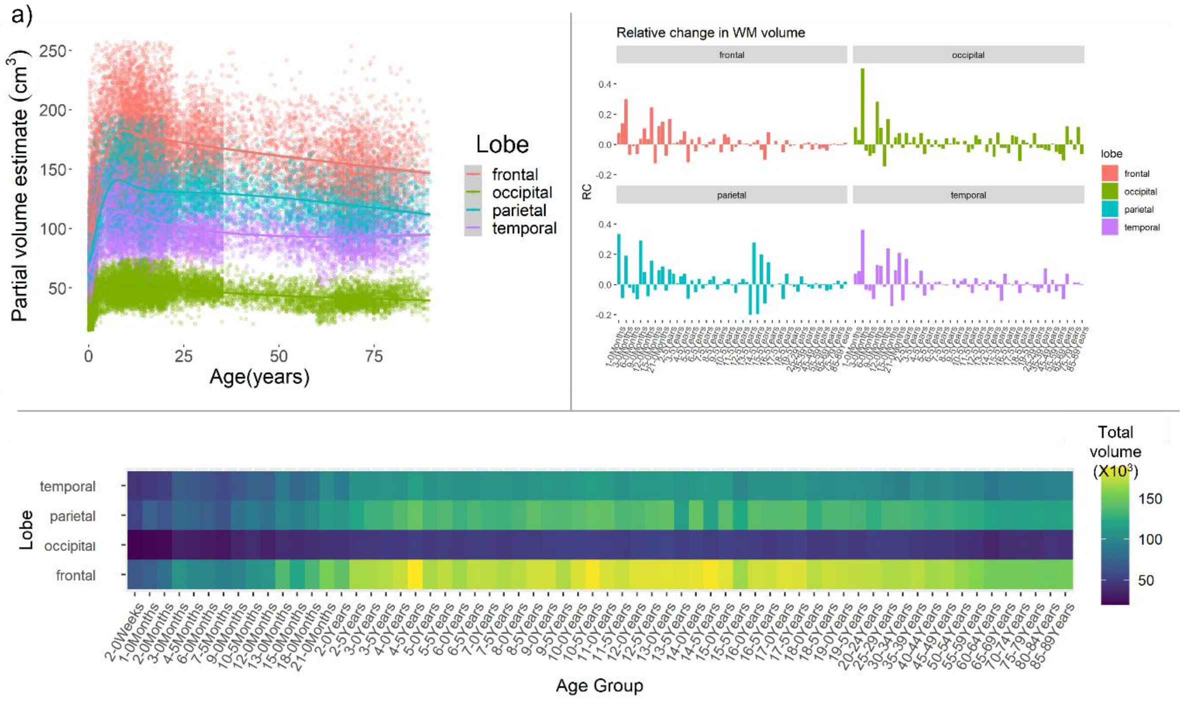

b)

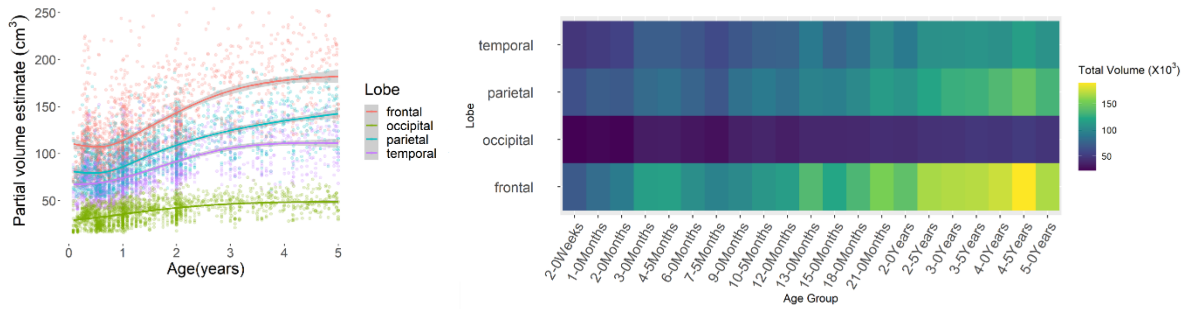

Supplement: S3 Fig — Panel a) Residual scores for the changes in WM across the lifespan for each lobar region (top left) and their relative change (top right). Heatmap of the total WM volume change for each age group and lobar region (bottom). Panel b) Residual scores of the WM changes (left) and heatmap of WM volume changes (right) for each lobar region for the first five years of life. (PDF) [file pone.0301520.s003.pdf]
