# Supplementary material for: White matter trajectories over the lifespan
Source: PLoS One. 2024 May 17;19(5):e0301520. doi: 10.1371/journal.pone.0301520 (PMC11101104; doi:10.1371/journal.pone.0301520)

Supplementary Figure S4. Residual scores for the changes in WM partial volume cross the lifespan as a function of the database (left panel). Relative change in WM partial volume across all age groups (right panel).

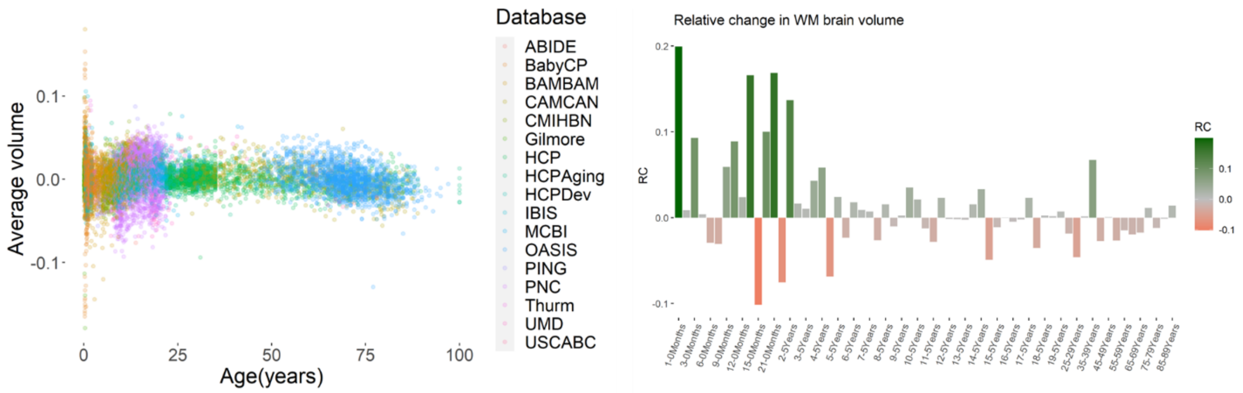

Supplement: S4 Fig — Residual scores for the changes in WM partial volume cross the lifespan as a function of the database (left panel). Relative change in WM partial volume across all age groups (right panel). (PDF) [file pone.0301520.s004.pdf]
