# Supplementary material for: White matter trajectories over the lifespan
Source: PLoS One. 2024 May 17;19(5):e0301520. doi: 10.1371/journal.pone.0301520 (PMC11101104; doi:10.1371/journal.pone.0301520)

Supplementary Figure S5. Axial view of the reconstructed WM tracts from representative infant and adult subjects, along with their sagittal views overlayed to the participant's T1 scan.

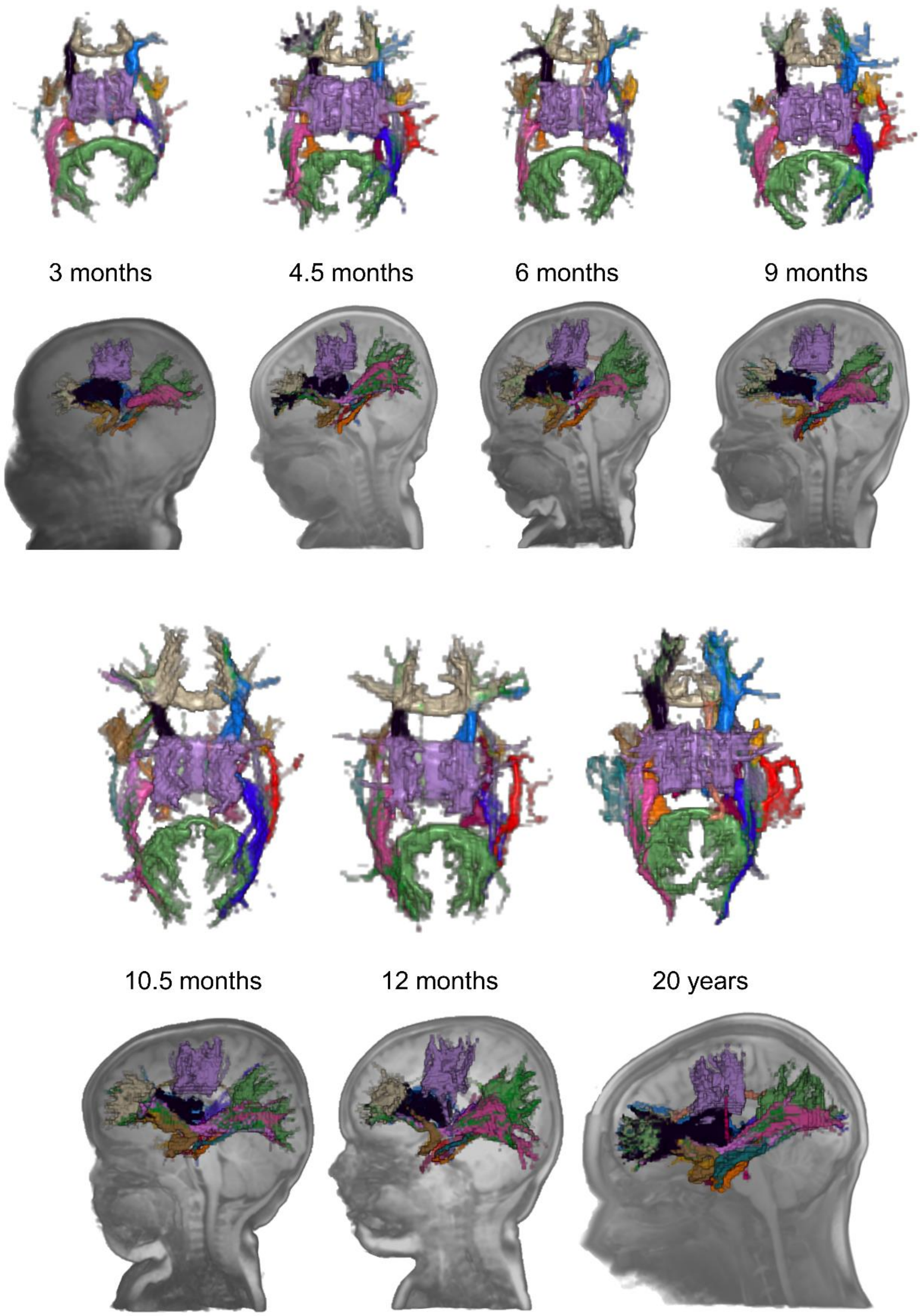

Supplement: S5 Fig — (PDF) [file pone.0301520.s005.pdf]
