# Supplementary material for: White matter trajectories over the lifespan
Source: PLoS One. 2024 May 17;19(5):e0301520. doi: 10.1371/journal.pone.0301520 (PMC11101104; doi:10.1371/journal.pone.0301520)
Supplement: S1 Table — (PDF) [file pone.0301520.s006.pdf]

Supplementary Table S1. Total number of volumes for each database.

Database	Age range	N
ABIDE	7-19 years	172
BAMBAM	2 months - 14 years	1171
CAMCAN	18 - 89 years	604
CMI-HBN	9 months - 24 years	674
Gilmore	2 weeks - 2 years	201
HCP		
Baby	2 weeks - 5 years	699
Developmental	8 - 24 years	654
Young adults	18.5 - 34 years	1031
Adults and Aging	35 - 89 years	694
IBIS	6 months - 2.5 years	300
MCBI	3 months - 69 years	703
OASIS	45 - 99 years	1611
PING	3 - 24 years	772
PNC	8 - 24 years	1415
Thurm	1 - 3 years	55
USCABC	20 - 79 years	60
