# Supplementary material for: White matter trajectories over the lifespan
Source: PLoS One. 2024 May 17;19(5):e0301520. doi: 10.1371/journal.pone.0301520 (PMC11101104; doi:10.1371/journal.pone.0301520)
Supplement: S2 Table — (PDF) [file pone.0301520.s007.pdf]

Supplementary Table S2. Variance and AIC values of each considered brain mesure for models with and without Database as random effect (RE)

**Measure**	**Database**	**AIC**	**RE intercept variance**	**RE residual variance**
GM_{norm}	no	-28285.13	N/A	N/A
GM_{norm}	yes	-29023.60	0.00121	0.00031
WM_{norm}	no	-29298.91	N/A	N/A
WM_{norm}	yes	-31377.55	0.00042	0.00038
FA	no	-20149.26	N/A	N/A
FA	yes	-29172.47	0.00135	0.00023
MD	no	-71662.77	N/A	N/A
MD	yes	-86565.62	$0.30X10^{-7}$	$0.28X10^{-8}$

There was a model improvement when Database was included as random factor, highlighting the increased variance produced by the different acquisition parameters of the studies included in the current work. Longitudinal and multi-center studies have become useful tools to analyze brain changes across a wide age and participant range. Nevertheless, several technical factors (e.g., MRI sequence parameters, operating system, software version, etc.) can influence the outcome of the study (Gronenschild et al., 2012) and contribute as an additional source of variance when integrating data from different sources. In the current study we further reduced such variability by excluding the diffusion analyses datasets that used single-shell acquisitions.
